# Socioeconomic Factors Influencing the Growth and Sustainability of the Village Movement

**DOI:** 10.1155/jare/5295292

**Published:** 2025-06-12

**Authors:** Yi-Ting Chiu

**Affiliations:** Schar School of Policy and Government, George Mason University, 3351 Fairfax Drive, Van Metre Hall, Arlington, Virginia 22201, USA

**Keywords:** aging in place, community development, community organizations, gerontology, mixed-methods, Village Movement

## Abstract

This study offers the first in-depth analysis of the Village Movement since its launch in 2002, examining the socioeconomic and institutional factors behind the growth and sustainability of Villages, community-based organizations that support aging in place. Using a mixed-methods approach, the research combines survey data, case studies, interviews, field observations, and regression analysis. Findings show that Villages are grassroots' responses to the breakdown of traditional social networks in modern, urbanized societies. They help rebuild community ties, reduce isolation, and provide older adults with both services and a renewed sense of purpose. Key drivers of Village development include human capital, civic engagement, spatial proximity, and support from existing organizations. While strong local networks can sometimes reduce the need for Villages, communities with looser ties often have greater motivation to establish them. The study also challenges the perception that Villages are expensive or exclusive. Many operate with low or no membership fees, and alternative models, such as affiliated or hub-and-spoke Villages, allow for growth in lower income and minority communities. Government support, while not essential, can complement Village efforts, particularly during crises such as the COVID-19 pandemic. Overall, the Village model proves adaptable and resilient, capable of filling service gaps while strengthening community cohesion. It offers a flexible, community-driven solution to the challenges of aging in a rapidly changing society.

## 1. Introduction

As the global population ages, the demand for sustainable, community-based aging solutions has become increasingly urgent. In the United States, the Village Movement, which began in 2002, represents a grassroots' response to this challenge by fostering community networks, volunteer engagement, and locally organized services that allow older adults aging in place [1, 2]. Different from assisted living and retirement communities, Villages do not need older people to move somewhere else to get services. Instead, the Villages are established and operated by the older adults who already live in the neighborhoods, to provide the nonprofessional services needed by themselves and their older neighbors, including driving, cleaning, house repairs, educational programs, and social events. By leveraging existing community relationships and social capital, Villages help older adults remain in their homes while staying socially connected and maintaining independence [3].

The flexibility of the Village model makes it adaptable to a variety of geographic, demographic, and socioeconomic conditions [4]. Some Villages are entirely volunteer-run, while others integrate paid staff, membership fees, and external grants to sustain operations. In addition, although operating independently, most Villages choose to participate in global and regional Village coalitions, such as the Village- to-Village network, so that they can network with each other to facilitate resource sharing, best practice dissemination, and policy advocacy [1]. Thus, the Villages not only help older residents aging in place but also improve community engagement and volunteerism. The Village has become a promising model for aging in place and community engagement. Today, there are more than 300 Villages operating in 41 states of United States, with additional Villages in Canada, Australia, and the Netherlands [5].

The Village model is valuable because, compared to other aging-in-place models, it depends less on government resources and is more adaptable to various geographic settings. While the Village movement is a type of social movement, it is also a good example of how, in a postindustrialized world, people at the local level cooperate to take on their common challenges. Despite the growing adoption of the Village model, existing research has largely focused on the impact of Villages on members' well-being, with limited attention given to the broader socioeconomic and institutional factors influencing Villages' formation and sustainability [2, 6]. While studies have demonstrated that Villages can enhance social connections, reduce isolation, and improve quality of life, the conditions that enable some communities to successfully establish and sustain Villages, while others do not remain underexplored [1, 7].

To address this gap, this study examines the socioeconomic, organizational, and institutional factors influencing the Village Movement. By integrating theoretical perspectives from social capital theory, community resilience theory, social entrepreneurship, and organizational network theory, this research provides a comprehensive framework for understanding the emergence and sustainability of Villages [8–11]. Furthermore, different from the previous studies, which solely relied on self-reported survey data, this research incorporated other forms of data and analysis such as interviews, field observations, and regression analyses. More importantly, the findings of these different research methods can support each other, so the reliability of this research was strengthened. Furthermore, as the older ethnic minority population is growing, a focus on Villages in communities of ethnic minorities or lower incomes helps to understand how the Village concept works in those communities. This study seeks to answer three central research questions: (Q1) Why have Villages started and grown in the last 2 decades? (Q2) Are certain neighborhoods with specific cultural, educational, or demographic features more likely to have a Village? (Q3) What factors influence the sustainability and long-term success of Villages?

Based on the questions outlined above, nine hypotheses have been established. The first hypothesis focuses on the concept of community resilience. As defined by Ganor and Ben-Lavy [12], community resilience refers to “the ability of individuals and communities to deal with a state of continuous long-term stress, the ability to find unknown inner strengths and resources to cope effectively, and the measure of adaptation and flexibility.” In this context, aging populations across neighborhoods in the United States can be viewed as communities facing crises driven by the broader aging trends in American society. The Health Department report indicates that the preolder population, aged 45–65, increased by 14.9% between 2005 and 2015 across the United States. ([13], p. 1). However, focusing on the age group from 55 to 64, William [14] found that this population has increased by nearly 50% in size from 2000 to 2010. The paper attributes this rapid growth to the baby boom generation.

The consequences of the growing aging population include loneliness, ageism, and the rising costs of care. The lack of adequate resources for older adults remains a pressing public policy issue in the United States. This challenge encompasses limited and often inadequate pension systems, higher healthcare costs compared to other nations, a shortage of affordable and age-friendly housing options, and difficulties in connecting older adults with essential health services [15–17]. Furthermore, as the aging population continues to grow in both size and proportion, the demand for social resources is expected to become even more widespread and strained. According to community resilience theory, when institutional responses fail to provide adequate support, affected communities often develop bottom-up solutions to address systemic gaps [9, 18]. The Village Movement exemplifies this adaptive response, as older adults have mobilized to create self-sustaining organizations that provide social, logistical, and emotional support. This aligns with social movement theory, which suggests that grassroots' mobilization emerges when communities experience structural strain and unmet social needs [19].

### 1.1. H1: The Crises Brought by the Growing Aging Population (in Terms of Amount and Percentage) Are Part of the Factors That Led to the Village Movement

The second hypothesis is based on the relationship between educational attainment, self-perceived capabilities, and social entrepreneurship. Social entrepreneurship refers to activities that prioritize social or environmental outcomes rather than profit maximization, often characterized by innovation in organizational models, processes, or services, as well as a strong emphasis on performance measurement and accountability [20]. Research suggests that individuals with higher educational levels and strong self-efficacy are more likely to engage in social entrepreneurship and community organizing [8, 21]. A key factor in the Village Movement is that it requires older adults to take the initiative in organizing, managing, and sustaining the community-based model for aging in place. Given that social entrepreneurship often emerges in response to gaps in public services, education plays a crucial role in equipping individuals with the skills necessary to develop, implement, and sustain these social enterprises. This directly contributes to the successful establishment of Villages.

Furthermore, education has been linked to a preference for autonomy in aging. Older adults with higher educational attainment are more inclined toward independent living rather than relying on traditional family structures for care. They tend to seek community-driven solutions such as Villages, which allow them to age in place while maintaining a sense of control over their lives. In addition, those with higher self-perceived capabilities are less likely to internalize self-stigma associated with aging. Instead, they feel more confident in tackling aging-related challenges proactively by forming and participating in Villages. Based on this hypothesis, the educational feature of the baby boom generation also contributes to the growth of Village. William [14] suggests that the preolder population is more educated and with more professional women than their previous generations.

### 1.2. H2: Higher Education Levels and Self-Perceived Capabilities of Older Adults Are Positively Associated With a Stronger Village Movement

The third hypothesis builds on literature regarding community organization and social capital. Social movements at the community level are often supported by strong networks, which facilitate resource sharing, information exchange, and collective action. The Village Movement aligns with this theoretical framework, as it relies heavily on social connections for recruitment, funding, and operational sustainability. Newman and Dale [22] suggest that social networks are composed of both bonding and bridging ties, each influencing resilience differently. Bonding ties, close-knit, inward-looking relationships, help sustain trust and mutual aid within a community, which may support the formation of Villages. However, communities with excessively strong bonding ties may feel less need to establish new organizations, as their social networks already provide sufficient support. Conversely, bridging ties, connections between diverse groups, enhances access to external resources, partnerships, and innovative solutions, making them critical for the sustainability and growth of Villages.

### 1.3. H3: Well-Connected Social Networks and Stronger Organizations Within and Across Neighborhoods Are Positively Associated With a Stronger Village Movement

The fourth hypothesis is about governmental activism. As Stephan et al. [23] mentioned, governmental activism would have two opposite impacts on social entrepreneurship: both the insufficiency or provisions of public resources could trigger social entrepreneurship activities. Thus, governmental activism could be benign or malignant to the Village Movement.

### 1.4. H4: Governmental Activism Is Positively Associated With a Stronger Village Movement

The fifth hypothesis links social movement theory and civic culture to the Village Movement, emphasizing that socially supportive cultures foster civic engagement, volunteerism, and collective problem-solving. The “New Netherland” region, known for strong civic participation, was an early adopter of the Village model [24]. Conversely, some North American regions are less conducive to social participation. Woodard [24] identifies the following: “Tidewater,” which values authority over public engagement; the “Deep South,” historically shaped by aristocratic privilege and racial hierarchy; and the “Far West,” where skepticism toward federal intervention limits civic activism. These cultural traits have contributed to lower levels of civic engagement in these regions.

### 1.5. H5: Socially Supportive Culture Is Positively Associated With a Stronger Village Movement

The sixth hypothesis examines the spatial dimensions of community engagement, emphasizing how geographic proximity influences social interactions and the formation of Villages. Research suggests an inverse relationship between spatial distance and the likelihood of friendship formation [25, 26], with closer living arrangements fostering stronger social connections, even across racial and generational lines.

### 1.6. H6: Accessibility and Spatial Proximity Are Positively Associated With a Stronger Village Movement

As for the impacts of income on the Village Movement, although joining Villages is a cheaper way to get nonprofessional services, Graham et al. [3] indicate that Villages predominately exist in non-Hispanic communities. These facts suggest that Villages might be more popular in middle or middle–high-income neighborhoods. Older people with very low incomes might be more likely to get help from charities or government subsidies and thus have weaker incentives to participate in grassroots' organizations to get help. Moreover, residents of lower income communities might need to spend most of the time making a living, and thus be less likely to be volunteers for Villages. In contrast, people with very high incomes would also be less interested in participating in Villages since paying for the services they need would not be a big issue. Therefore, the relationship between income and the strength of the Village Movement would be a concave curve.

### 1.7. H7: The Relationship Between Income and the Strength of the Village Movement Follows a Concave Curve. This Means the Village Model Would Be More Popular in Middle or Middle–High-Income Communities

The eighth hypothesis posits that Villages not only provide essential services but also serve as catalysts for fostering social bonds that sustain their long-term viability. Research on social capital suggests that participation in voluntary associations enhances civic skills, strengthens community ties, and promotes engagement [9, 27, 28]. Villages, as social infrastructures, facilitate intergenerational relationships and broader community involvement, reinforcing their sustainability by attracting more members, volunteers, and funding. By fostering new social connections, including cross-generational relationships, through service delivery and communal activities, Villages increase the density and cohesion of local social networks. This growing interconnectedness strengthens the fabric of the community. Enhanced social ties contribute to Village sustainability by mobilizing resources, encouraging participation, and deepening the sense of belonging among members.

### 1.8. H8: The Village Would Create Social Connections, and These Connections Would Help to Maintain the Village's Operation

The final hypothesis explores the psychological and emotional benefits of Village participation. In postindustrialized societies, where productivity is highly valued, many older adults experience a diminished sense of societal worth as they age. Villages provide an avenue for them to regain a sense of purpose by contributing to their communities, fostering a renewed sense of agency and belonging. Research suggests that community engagement enhances self-worth, mastery, and social connectedness, making participation in Villages not only a practical choice but also an emotionally fulfilling one. The opportunity to support others fosters a positive cycle of engagement, which strengthens the sustainability of the Village model over time. Studies indicate that older adults involved in community-driven organizations report stronger social connections, higher neighborhood satisfaction, and reduced feelings of insecurity [29, 30]. Furthermore, community engagement has been shown to provide a sense of purpose, increased self-worth, and cognitive stimulation, reinforcing the overall well-being of older adults [31]. By fostering meaningful participation, Villages empower older adults to remain active, engaged, and valued members of their communities, ultimately improving their well-being while ensuring the longevity of the Village model.

### 1.9. H9: For Older People, the Sense of Value Creation and Self-Fulfillment Obtained by Operating Villages Would Be a Reason for Participating in the Village Movement

Table 1 presents the research questions, corresponding hypotheses, and the methods used to test them.

## 2. Materials and Methods

This research applied the mixed-methods approach for data collection and analysis [32]. According to Dr. Udo Kelle, mixed methods involve “the combination of different qualitative and quantitative methods of data collection and data analysis in one empirical research project” [32]. The literature suggests two key purposes for using mixed methods [32, 33]: The first is to ensure validity: by combining methods with different strengths and limitations, researchers can test whether findings from one approach are supported by another, reducing the risk of bias. The second is enhancing understanding: since qualitative and quantitative methods offer different perspectives, their integration provides a more comprehensive view of the studied phenomenon. Therefore, three reasons make the mixed-methods approach superior to alternative approaches for carrying out this research. First, the advantage of the mixed-methods approach is its ability to cross-validate findings, making it superior to a single-method approach. Quantitative data, such as surveys and statistical models, offer measurable trends, but they may lack context and fail to explain why patterns exist. Conversely, qualitative methods, such as interviews and case studies, provide rich, detailed insights but may lack generalizability. By integrating both, this research compensates for the weaknesses of each approach while leveraging their strengths. For instance, statistical analysis can reveal correlations between Village participation and social capital, while interviews can clarify the lived experiences of Village members and their motivations. This methodological triangulation ensures that conclusions are not skewed by the limitations of any single method. Second, the Village Movement is a newly emerging phenomenon with limited prior research. The factors that drive its development include cultural norms, social networks, and economic conditions, each of which requires different analytical approaches. Quantitative methods help identify broad patterns in Village formation, membership demographics, and resource distribution. In contrast, qualitative methods offer deeper insight into why individuals participate, how Villages evolve, and the social dynamics at play. If this research had used only one method, it would have missed critical dimensions of the phenomenon. Mixed methods allow for a fuller picture by examining both structural trends and individual experiences, ensuring a more nuanced understanding of how and why Villages sustain themselves. The last key advantage of mixed methods is its adaptability. Greene et al. [34] note that sequential analyses allow one method to inform the next. This iterative process enhanced the study's depth and precision, making it superior to a static single-method approach, which would have lacked the ability to refine the research focus dynamically.

The research methods applied in this study are Village survey, Village case study, and regression analysis. The three methods were carried out interchangeably depending on practical situations (such as whether the interviewees responded to the interview requests), and, during the process of data collection and analysis, the information obtained from each of the main methods was used to help the design of other research methods. For example, the interview answers helped to design the survey questionnaires; the analyses of the Village case study were also used to select the independent variables of the regression analysis and to explain the result of the regression analysis. More details about the design of each research method are given in the following paragraph. In addition, for the data collection process that involved human participants, the procedures and relevant documents were approved by George Mason University's Institutional Review Board. All participants also provided written consent by signing the informed consent forms.

### 2.1. Survey

The Village survey is to understand the general information of all the Villages in the United States, especially about the Villages' establishment and operation. The Villages that meet the following criteria are included in the survey: (1) Providing services before January 1, 2020, (2) being in the United States, and (3) identifying themselves as a Village. The list of the Villages is based on the lists given by the largest Village global and regional coalitions, Village-to-Village (V-to-V) Network and Washington Area Villages Exchange (WAVE).^1^ The survey sent out the questionnaires and recruited responses from August 24, 2020, to March 2021. Moreover, before the survey began, a pilot survey was conducted from Feb 1, 2020 to May 25, 2020, and responses from five Villages were obtained. Then, the survey questionnaires were revised according to the responses and feedbacks from the Villages participating in the pilot survey. As a result, among 317 Villages that were open, 120 of them responded to the survey with a responding rate of about 37.9%. The survey system “RedCap” was used to send out the questionnaires and collect responses.

### 2.2. Case Studies

The Village case study serves as a complement to the regression analysis for testing the research hypothesis. Compared to the regression analysis, which is variable-based, the case study would have more depth in understanding and be better for determining causal mechanisms and exploring the factors behind a phenomenon. Therefore, the case study is more applicable to exploratory theory-generating research. Since various theories could suggest the possible factors behind the Village Movement, the case study would help researchers understand the interactions of those theories. Also, since some answers to the research questions are not contained in the literature, answering the questions requires an exploratory methodology and the case study helps to do that. Therefore, this research applied the case study method by choosing several Villages and studying how they are established and operated. Two case study approaches, causal-process tracing and congruence analysis, were applied ([35], p. 27). The former approach focuses on the causal mechanisms and conditions of a Village's development; the latter approach emphasizes understanding the interactions of competing and complementary theories which could explain the Village Movement.

The selection of case Villages follows a combination of exceptional cases and most likely cases, considering both theoretical and practical factors. According to Gerring [36], exceptional cases are those that significantly deviate from expected trends or theoretical predictions. These outliers help explore how Villages emerge in less favorable conditions. In contrast, most likely cases occur under conditions that strongly support Village formation. This study includes one most likely case as a control for comparison.  Table 2 presents the criteria used to select cases, ensuring a diverse and representative sample. The following are the key selecting factors and the corresponding research hypothesis tested:

• Geographic setting: Cases were chosen from urban, suburban, and rural areas to examine the impact of spatial dynamics on Village formation (H6). Since all Villages are located in the Metropolitan Statistical Areas delineated by the United States' Office of Management and Budget, self-identification in the Village survey is used to see whether the case Villages are located in rural areas.
• Income and educational levels: Cases were selected from different socioeconomic backgrounds to explore the relationship between economic status and Village sustainability (H2 and H7).
• Community participation culture: Cases were selected from regions with both high and low levels of civic engagement to evaluate the impact of cultural norms on Village development (H5). The geographic distribution of the region is based on the data and definition given by Woodard [24].
• Ethnic composition: Given the predominance of Villages in non-Hispanic white communities, cases including higher proportions of ethnic minorities were chosen to assess the cultural adaptability of the Village model [7].

Beyond research hypotheses, interviews revealed that different Village operating models play a crucial role in how Villages adapt to local conditions. As a result, case selection also considered the following operational models, categorized in Table 3.

• Standalone Villages: Independently operated Villages that rely on membership fees, donations, and volunteer networks.
• Hub-and-spoke Villages: A central organization provides administrative support to multiple smaller Villages, reducing operational burdens at the local level.
• Affiliated Villages: Villages established under the auspices of existing organizations (e.g., churches, senior centers, or local governments) that benefit from institutional support.

Furthermore, Table 4 shows the data source of Village case studies. The data analysis process is based on Miles et al. ([37]; Chapter 4) and Saldaña ([38]; p. 68). First, codes are assigned to various parts of data; according to Miles et al. [37], “codes are labels that assign symbolic meaning to the descriptive or inferential information compiled during a study.” This process is called “coding.” Then, codes with similar meanings were merged. There are 203 codes after the merge. Next, these 203 codes were categorized into 18 code groups. These code groups are not mutually exclusive; in other words, one code could be categorized into more than one group. After that, the relationships between different codes and documents were observed. Memos were also written throughout the whole process of the analysis to note findings and analysis. Themes emerged during this categorizing, observing, and memo-writing process. 2 Finally, according to those themes identified by this study, an outline of the analysis results was written and then the full text of the result was written. The causal-process tracing method was also applied to analyze the founding processes of the case Villages ([35], p. 27). ATLAS.ti was chosen as the analysis software for analyzing the qualitative data. While using software is not necessary for qualitative analysis, using software such as ATLAS.ti helps to effectively create and categorize codes, retrieve transcripts by specific codes, find which transcripts contain certain codes, and organize memos. The software could also be used to organize code and visualize the relationships among codes.

### 2.3. Regression Analysis

The research period, 2002 to 2020, is from the year when the first Village opened to the year when this study conducted the Village survey. 3 For observation and dependent variables, this research used areal data at the county level and county–subdivision level to carry out the regression analysis. That is to say, each county or county subdivision was taken as a single observation or analysis unit.

Table 5 gives the total number of counties and county subdivisions in the United States, the reasons for selecting the analysis units, and the sources of the corresponding shapefile data chosen by this study. Moreover, the dependent variable is, in the geographic analysis unit, the number of Villages opening during the survey period, August 24, 2020, to March 2021. For example, during the survey period, if a county has two Villages, then, for that county observation, the value of the dependent valuable is two. This research used two types of dependent variables: the number of opening Villages and the total Villages. In addition to opening Villages, the total Villages also include five Villages that closed during the pandemic and 43 Villages which are in development. Thus, the regression models using the total Villages as the independent variables could suggest future trends for the Village Movement.

**Figure 1:**
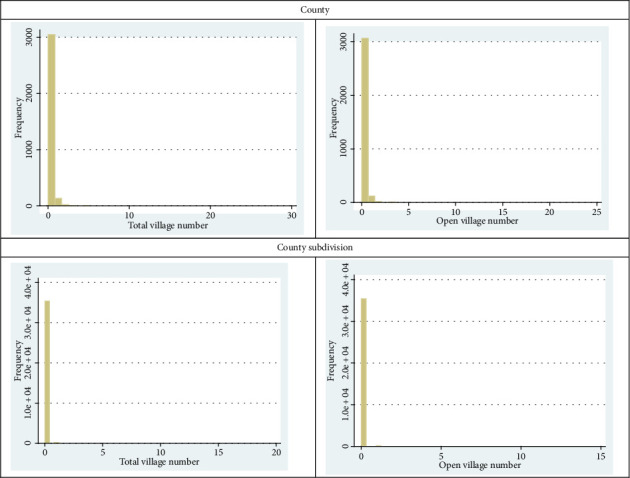
Distribution of independent variables at the county and county–subdivision levels (The *X* axis shows the number of Villages within the number of counties or county subdivisions on the *Y* axis; the *Y* axis is the number of counties or county subdivisions containing *X* number of Villages).

The zero-inflated negative binomial (ZINB) and zero-inflated Poisson (ZIP) models were chosen to analyze factors influencing the emergence and growth of Villages. This decision is based on the nature of the dependent variable, the number of Villages within geographic units (counties or subdivisions), which exhibits an excessive number of zero counts. As shown in Figure 1 in the Appendices, many geographic units have no Villages. Traditional count models such as Poisson and negative binomial regression assume that zeros result solely from the underlying count process, making them unsuitable for this data structure. These models were initially considered but failed to account for the excess zeros, leading to poor fit and potential bias in coefficient estimates. To validate the choice of zero-inflated models, Vuong's test was conducted to compare the standard Poisson and negative binomial models with their zero-inflated counterparts. As shown in Table 6, Vuong's test statistics are significant for both comparisons, confirming that the zero-inflated models provide a significantly better fit. The results indicate that a separate zero-generating process is necessary for accurately modeling the data. Given these findings, the zero-inflated models provide a more appropriate framework for distinguishing between geographic units where Villages are unlikely to form and those where socioeconomic factors actively influence Village establishment. By employing these models, we improve the robustness of our analysis and enhance the accuracy of our inferences regarding the drivers of the Village Movement.

Based on the zip code of the office address provided by Villages, this study used the “Geocode” function of the ArcGIS software and the 2019 Census 5-Digit ZIP Code Tabulation Area shapefile to get the Village point data in shapefile format. 4 Therefore, in the shapefile of the Village point data, the centroids of the zip code areas are taken as the Village locations. Then, this study used the “Spatial Join” function in the software to obtain the Village number in each county and county subdivision. The spatial join matching option chosen was “completely contain.”5 As for independent variables, many socioeconomic figures have changed during the 200–2020 research period; for selecting independent variables, this research chose the data year closest to the year 2011 to get the average socioeconomic feature of the research period.  Table 7 lists all the independent variables used in the regression analyses, including their tested hypothesis, expected direction, unit, year, and sources. Two types of variables, income and social capital, were taken as square values to capture the nonlinear relationship. The nonlinear relationship between income and the Village number is explained in Hypothesis 7. For social capital, a nonlinear relationship was found in the analysis of the qualitative data: while certain levels of social connections and networks could be helpful in establishing Villages, very strong local social networks could reduce people's incentives to start Villages. Moreover, the spatial effect variable is to see whether the geographic units with a higher number of Villages are more likely to be located closer to each other. Since the spatial analysis model used by this research failed to converge, this variable was used instead. It measures the number of neighboring spatial units having Villages. Also, when this study ran regression analyses, some variables were dropped due to high correlations.  Table 8 lists those selected and unselected variables, and their correlation parameters.

## 3. Results and Discussion6

This section examines how survey data and case studies contribute to understanding the growth and sustainability of the Village Movement. The findings help test the research hypotheses by identifying patterns and contextual factors influencing Village development.

### 3.1. Survey and Case Studies

**Figure 2:**
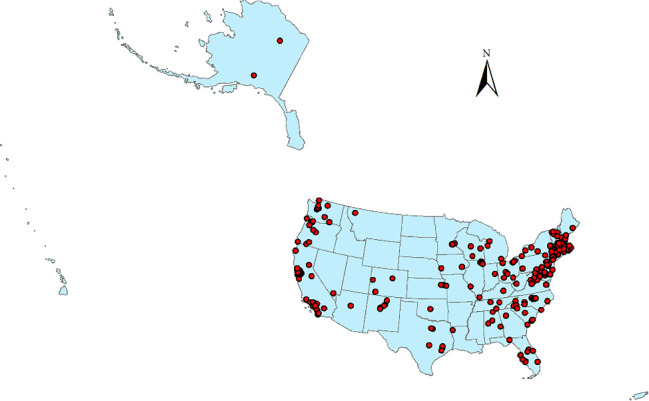
Geographic distribution of Villages.

**Figure 3:**
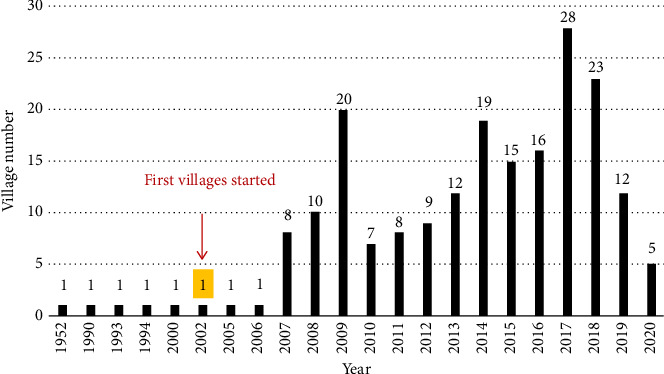
Year in which Villages started to provide services (total Village number: 200) (note: the first Village, Beacon Hill Village, has provided services since 2002).

Table 9 summarizes the operating status of Villages across the United States. Of the 364 identified Villages, 317 were operational, 43 were in development, and four had temporarily closed due to COVID-19. Among the active Villages, 120 responded to the survey, yielding a 38% participation rate. However, response rates varied for different survey topics, meaning some analyses included fewer than 120 Villages.  Figure 2 illustrates the geographic distribution of Villages, while Figure 3 presents the founding years of Villages. Although the first Village was established in 2002, some Villages reported earlier founding years. This discrepancy is due to Villages that evolved from pre-existing organizations, such as senior centers, which recorded their parent organization's founding date rather than their own.

#### 3.1.1. Uniqueness of the Village Model (H8 and H9)

Unlike traditional service organizations, Villages are community-driven, meaning that older residents establish and manage them based on local needs. This flexibility allows each Village to adapt to its specific community, a trait emphasized by an interviewee who stated, “*…one of the sayings in the Village community is when you've seen one Village, you've seen one Village. Every Village is different because the focus is on your people, on what they need.”* This adaptability of Villages was particularly evident during the COVID-19 pandemic. Many Villages quickly pivoted their services to meet emerging needs, a level of agility that larger organizations struggled to achieve. The director of the Department of Aging and Community Living in Washington, District of Columbia (DC), observed, *“We found during the pandemic that it's very hard to shift those models when you need the organizations shifted, but the most quickly and the most successfully shifted we found were Villages* [40]*, Day 3*, 2021.”

Building social networks within communities is particularly valuable in modern society, in which people seldom stay in the same place throughout their lifetime and thus the community networks are fewer and looser. By receiving services, volunteering, and going to Village events and programs, social networks are established. A Village volunteer who had volunteered for other organizations indicates that “(being a Village volunteer creates an) expanding circle of friends in the neighborhood. Being a volunteer in other places I would not have such chances.” Establishing social networks not only reduces social isolation and creates a sense of community but also increases the community's resilience. As mentioned in the literature review, greater community resilience could be built by ties between family and friends within the communities working with the external networks that channel external resources into the communities [41]. As suggested by a Village coordinator, the community networks and social connections created by Villages might be even more valuable than the services they provide.

There are Villages in Montgomery County and outside the county that don't even offer services. They are just creating local networks of social connections. It is recognizing the value of being a community that drives people, not necessarily the concrete service they might be able to get. The difference between getting a ride from Uber, other than the fact that you're paying for it, and getting a volunteer ride from a neighbor is that level of caring and support you get. It's not just the service, it's how you're getting it and from who you're getting it that makes the difference. It's really important to differentiate (between) regular social service organizations and Villages. They might look on the outside and the same, they might be judged and evaluated by traditional concepts but they're dramatically different. Quotation 1

The social networks also build trust among Village participants, and trust is crucial to make people in need actively seek help. A Village participant cited the value of trust created by Villages during the COVID-19 outbreak as

I think one of the things that we've learned is that older people are looking for a trusted advocate. I know for our older adults, they find that in Village, they'll call Village before they'll call anyone else. I think that you develop that relationship, you develop that trust, and you give them that stability, that person or that organization that cares, and they know you care. They have that sense of somebody's there for me [42], V-to-V Network*, Tuesday, October 6*, 2020. Quotation 2

The above quotations show the causation that trusts have been established through the social networks built by Villages as the members first seek help from the Village management and even other members when they are in need. A Village management person even found that their members prefer to ask for help through the social networks they built by participating in the Village, rather than directly asking the Village management for help. Village members are not just service recipients; they also contribute to the community. For instance, one member with mobility impairments, who relied on driving services, actively participated by creating birthday cards for fellow members. These findings demonstrate that Villages not only provide essential services but also foster social connections and trust among older adults, which aligns with Hypothesis 8. The adaptability of the Village model, as seen during the COVID-19 pandemic, allowed Villages to rapidly pivot services to meet members' needs, reinforcing the idea that community networks contribute to Village sustainability. In addition, Villages build social networks that reduce isolation and increase community resilience, supporting the idea that these connections help maintain Village operations. The evidence also supports Hypothesis 9, which argues that the sense of value creation and self-fulfillment obtained by operating Villages encourages older adults to participate in the movement. The findings illustrate how members are not just service recipients but also contributors, such as a member with mobility impairments who made birthday cards for others. Quotation 2 suggests that members feel a sense of belonging and purpose, reinforcing Hypothesis 9. Moreover, the differentiation between receiving services from a volunteer neighbor versus a paid service provider highlights the deeper social and emotional connections that Villages offer, further validating Hypothesis 9.

More importantly, while the case study analysis identified the factors causing Villages to be established, these factors are neither necessary nor sufficient conditions for establishing a Village. Some factors, such as social networks, could even be created by Village founders and participants themselves. The services and operating model of Villages could also be changed or recreated according to the needs of Village participants. Therefore, in addition to explaining how the various factors work during the Village founding and operating process, the following analysis also includes how it might work when a particular factor is absent.

#### 3.1.2. Human Capital (H2)

Human capital plays a positive role in establishing Villages, especially for those Villages founded earlier. Human capital supports both the motivations and abilities to establish a Village. For motivations, retired people with higher human capital tend to have more need to participate in intelligence-stimulating activities, and most senior-service organizations do not provide such activities. Thus, these people would need an organization such as the Village through which they could carry out activities they enjoy. A Village participant in the DC metropolitan area used the high level of human capital to explain why the region has more Villages.

Most of the people around here have worked for all kinds of things, with the World Bank or the IMF or the federal government or members of Congress. Those were very stimulating jobs and when they retired, it was a big shock, that they weren't doing anything that was as interesting as what they had done. Village provides a new community of pretty interesting people, very stimulating people and they got in Dupont Circle, the focus is on creating this community of interesting people and connecting people within it. Quotation 3

In addition to motivation, human capital aids in the ability to create a Village, since founding and running a nonprofit organization such as a Village need professional knowledge and skills, such as organizational management and fundraising. The working experiences of the first Village's founders also suggest the importance of human capital during the early stage: they included seasonal fundraisers and donors, civic leaders, self-employed professionals, retired corporate officers, and an artist [43]. The residents of the first Village are also well-educated [43]. This finding confirms Hypothesis 2.

Yet, although relevant professional skills could help to start and operate Villages, as more Villages are established and various Village operating models are invented, the importance of participants' human capital declines. Through Village coalitions, older Villages shared their experiences and documents with newer Villages. The coalitions also provide workshops to teach some Village operating skills such as grant writing. The details of Village coalitions' impacts are given in the following sections. In addition to Village coalitions, some Village operating models, such as the hub-and-spoke model, also make Villages rely less on their participants' human capital. Those Village operating models are illustrated in Section 3.1.10.

#### 3.1.3. Social Networks (H3 and H8)

Although interpersonal social networks can help to establish Villages, more loose and fragmented social networks could also provide the motivation to participate in Villages. These findings also reveal a **key deviation from Hypothesis 3.** Compared to contemporary older people, older people in the 1980s and 1990s have more and stronger connections to family, friends, and organizations in their living areas. Not only do they have more family around their neighborhood, but those families, especially female family members, are also more willing to take care of them, as described by one interviewee as

My parents were the generation having children after WWII and generally they, and certainly their parents, had more children than today's parents. In addition, they generally had more siblings than today's parents I think and the women were generally homemakers. Also, since they were not as mobile career-wise (nor were their husbands) as people today are, they had a more stable and long-existing circle of people such as siblings, long-term friends, and children that they could rely on nearby. That is not so much the case today.

Finally, I think female relatives were more open to taking in a close relative and as caregivers, they were more engaged in the job and paid more attention to their charge as far as their social needs were concerned. My mother moved my grandmother in with her when she became ill and unable to live independently. My great aunt cared for her sister. Another aunt took care of her sister who lived next door and when the weaker sister died, another sister moved in right after her and her sister helped her as well. This kind of commitment is not common today it seems to me. Quotation 4

The above quotation also confirms what is suggested in the literature that the baby boomer generation would have a shriveled social network [44]. Another interviewee also said that, in the DC district, many single women who are retired professionals helped to build Capitol Hill Village because they wanted the Village as their future safety net. In addition to fewer connections to their family and friends, the older generation today is less likely to participate in local faith-based or other organizations. In fact, the role that Villages play is similar to the faith-based community center in a traditional society. The traditional faith-based community centers provided social spaces, institutions, and activities; people could go there to make social connections and look for help. However, nowadays, fewer people participate in their local faith-based institutions. For example, while Christianity has been the most popular religion in the United States, the percentage of adults with church membership has declined steadily [45]. Even among those people going to the church, fewer of them go to the church near their houses, especially people in urban or suburban areas.

**Figure 4:**
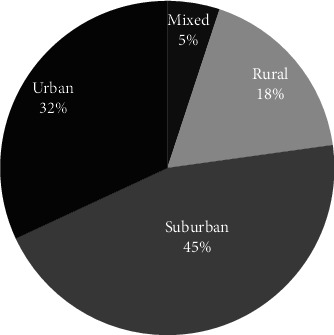
Geographic setting of Villages (total Village number: 197).

The two reasons mentioned above could also explain why Villages are more likely located in more urban areas, as Figure 4 shows. In rural areas, people are more likely to have family nearby, and more churches there still have local bases. In addition, while making new friends or plugging into the community are important motivations for participating in Villages, an opposite example also shows that strong social networks would cause a disincentive for participating in Villages. One interviewee is a community leader in Arlington County, Virginia. Although the community is located within the service area of Arlington Neighborhood Village, most of the community residents do not join that Village. This is because the community residents could get what they need through their existing community social networks. That community also has a local church that plays the role of the community center. The only exception known by the interviewee is a lady who moved into the community from other states after retirement. Since being newer to the community, she does not have strong community social networks as other residents do. Although she is highly involved in the local church, the church does not provide the full extent of services that the Arlington Neighborhood Village does. Therefore, she joined the Village. In contrast, a Village founder whose Village is in a popular retirement area said that some of the members are new residents and they join the Village for making new friends. An interviewee from another Village also said, “For our village, people join way before they might need services, and they join for social reasons.”

Although very strong community social networks might be a disincentive for participating in Villages, certain existing community social networks and associations do help to establish a Village. This finding supports Hypothesis 3. While Village coordinators suggest that a startup group composed of 3–4 people is adequate for starting a Village, the startup groups of some cases of Villages came from circles of friends within the communities. Indeed, the birth of the Village concept was from grassroots' conversations among friends within the neighborhood [46]. Moreover, while many Villages started from a Village open house introducing the Village concept, it would be helpful if people could bring their local friends to the open house. Some interviewed Village volunteers were also recruited by someone they know who participated in Villages. Furthermore, the promotion of the Village concept also relies highly on social networks across the communities. Since the Village concept is new and easy to be confused with other organizations also called “village,” for hearing about and figuring out what Villages are, it is important to know someone who understands the Village concept.

While social networks would be helpful in promoting the Villager concept and starting a Village, new social networks could also be created during the founding process. This finding supports Research Hypothesis 8. A case Village, My Glacier Village, had few members who already knew other members before they joined the Villages. Yet, after the members joined the Village, new social networks were formed, which sustain the Village. However, although new residents could also help to build a Village, stable residency within communities is better than a transient one. To illustrate, new residents going to stay for the next 10 years would have more chances to form new social networks than those for the next 2 years.

#### 3.1.4. Interactions With Other Organizations (H3)

**Figure 5:**
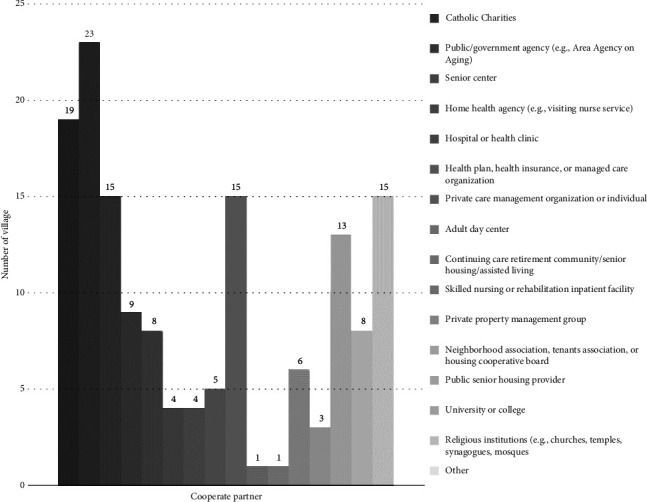
Types of organizations Village has a formal collaboration or partnership arrangement with (total participating Villages: 120).

While Figure 5 shows the types of organizations that Villages have formal partnerships with, the Village case studies explore the content of these partnerships and other kinds of interactions between Villages and these types of organizations. The study results show that, being small community organizations, all the case Villages got some kind of support from other organizations during the founding processes. It is also very common for Villages to collaborate with organizations of different specialties so that Villages could obtain more resources to operate Villages and benefit their members. This finding confirms Hypothesis 3 that stronger organizations within and across neighborhoods are beneficial to Village development.

##### 3.1.4.1. Other Villages and Village Coalitions

**Figure 6:**
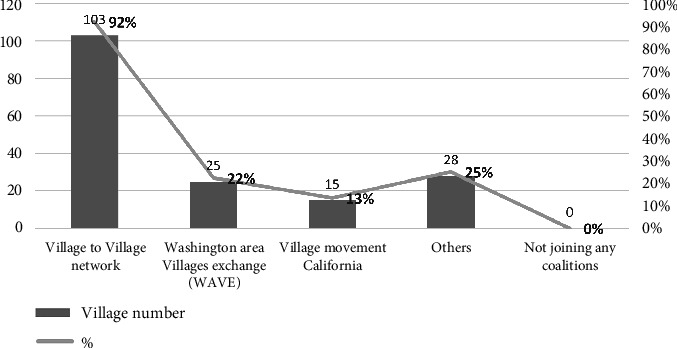
Village coalition participation (total: 112 Villages).

Among those other organizations, other Villages or Village coalitions might be the first type of organization one needs to ask for help from when starting a new Village. In some cases, Villages have consulted one or several Villages in nearby areas during their founding process. The founder of Golden Gate Village even had served other Villages before starting her own Village. Furthermore, Village coalitions might be a more important source of experiences for startup Villages. Through participating in the Village coalition, Villages network with each other to share resources and experiences.  Figure 6 shows that most of the Villages joined the global Village coalition, V-to-V Network, while some Villages also joined their local Village coalitions simultaneously.

Serving as platforms, Village coalitions connect to many Villages and their experiences, so more people could learn about the Village model. One interviewed Village founder said that before knowing the Village model, she was already very interested in the concept of working with neighbors to take care of older people in the community. She failed to realize the idea until she got information from the Village global coalition, as shown in Quotation 5.

Some years ago, I'm very interested in the concept of finding ways to work together with neighbors to take care of people as they age. I had tried to do this with just some of the few people I knew. We worked on a little bit about 10 years ago, but we realized it was just too difficult. We didn't have the tools. Somehow in an article or something, I ran across an article about V-to-V Network and that's the national organization. I looked at their website. I think I may have called one of the other Villages. I'm not sure, but anyhow, I realized that there was a way to do it, we would have support. Quotation 5

While there are many local Village coalitions, and also a global Village coalition for all Villages, among the case Villages whose location would allow them to join both, almost all of them say the local coalitions are more helpful than the global one. Several reasons could explain this preference: For the local Village coalitions, their member Villages are easier to get together physically, and the situations faced by the member Villages, such as regulations or grant opportunities, are also more similar. It is also easier for the local coalitions to better understand their Village members. An interviewee who leads a Village with special operating models mentions they have difficulties explaining to the global coalitions about their operating model. Thus, compared to the global Village coalitions, the local ones have a more connected and closer network. In addition, the membership fees for local coalitions are generally lower than that of the global ones.

Due to the above reasons, smaller or newer Villages only join the local coalitions if they need to make a choice due to limited resources. For example, while, on the list of the global Village coalition, there are around 35 Villages in Washington DC's metropolitan area, the list of the local coalition shows the area has about 75 Villages. Yet, according to an interviewee who sat on the coalition's board, among those 75 Villages, only about half of them substantially operate and pay the coalition's annual membership dues, which are $35. In contrast, the cheapest membership dues for the global coalition is $175. 7 However, although generally, the services of the local Village coalitions are more helpful, those provided by the global coalition are still valuable, especially for those Villages located in areas with few other Villages.

Furthermore, while most case Villages say they join Village coalitions for networking and educational purposes, there are exceptions. In one case, Village joined the global coalition membership for promotional purposes. This Village wants to ensure its name and contact information are on the coalition list so it could have more exposure to its potential members. Another exception is a coalition formed by the 13 Villages in Washington DC. These Villages not only shared experiences and data with each other but also collectively applied and managed the usage and distribution of the grant funds from the DC district government [40]. This collective application could increase the expected impacts of the grant application project and thus increase the chances of getting the grant. One reason for the success of this collaboration is that there is a sufficient number of Villages located in the same administrative area.

##### 3.1.4.2. Other Organizations

**Figure 7:**
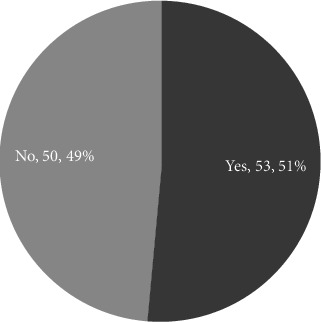
Whether Villages have formal collaborations or partnership arrangements (e.g., a contract, written MOU, or similar arrangement).

**Figure 8:**
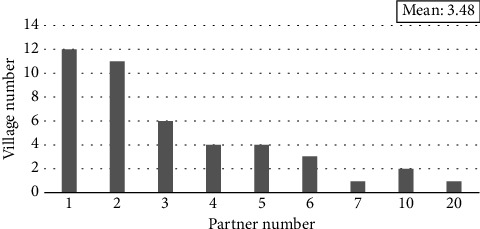
Partner number of Villages with formal collaborations or partnership arrangements (total participating Villages: 44).

The invention of the Village concept drew on resources from other local organizations. Located at Beacon Hill, Boston City, Massachusetts, the birth of the first Village benefited from the rich mix of professional and academic institutions in the area. During the founding process of the first Village, the founders developed a partnership with the local organizations. These organizations include a hospital, nonprofits running a retirement home and an Alzheimer's facility, and the Community Action Program of Harvard Business School. The first Village founders discussed the strengths and weaknesses of the Village concept with the experts from the above partner organizations. These partners also provided the Village with other resources such as free physical space for 1 year. More importantly, the partnerships increased the Village's credibility so that potential donors and members were more confident in the newborn Village. 8 This experience could explain why people like the interviewee who gave Quotation 5 failed to develop the Village model: since that interviewee lives in a suburban area, she has much fewer available resources from local organizations. Figures 5, 7, and 8 give the percentage, partner number, and partner types that the survey-participating Village has a partnership with other organization. These figures show that more than half of the Villages have partnerships with other organizations.

Similar to the experiences of the first Village, many Villages benefited during their founding processes from other organizations in the same communities. Sometimes these organizations, such as the homeowner association, provided opportunities and platforms to promote the Village concept to the local residents. For example, in some cases, Village founders presented at the gatherings of these organizations or issued an article or ad in the community newsletters, so that more residents learned about Villages. A case Village was even started as a program of a residential association and then became an independent Village [47]. Moreover, the existing organizations in the communities develop social networks and leadership that support the Village's establishment. The first Village also suggests that experiences in operating neighborhood organizations would be helpful to start a Village ([43], p. 4). Two case Villages said that their neighborhoods already had various community organizations, such as book clubs, before their Villages were established.

Villages also collaborate with local organizations in other forms. Some Villages cooperate with food pantries or grocery stores to deliver necessities to their members, some work with senior centers to train volunteers, some invited speakers from nearby universities to give talks on health-related topics, some get donations or member discounts from local businesses, and some applied for grants from local foundations. Moreover, local media is an important channel to promote Villages. After reading an article introducing the Village concept in a local newspaper, an interviewee founded the first Village in Montana. After a local TV station reported the grocery-delivery program provided by a case Village during the COVID-19 outbreak, many local older residents joined Village, and its membership doubled after the peak of the pandemic. In addition, in more than one case, the Village participated in their regional coalitions of health or older adult services providers. These coalitions serve more than one county, aiming to improve the health and well-being of residents. The coalition members, composed of nongovernmental organizations and governmental agencies, meet every month to share information and work together on some projects.

In addition, while fewer faith-based organizations play the role of community centers, as discussed in Section 3.1.1, many local faith-based organizations still help the development of Villages. As shown in Figure 5, the faith-based organization is still a type of organization that many Villages have partnerships with. The office space of one case Village is provided by a local church [47]. In some other cases, Villages were even developed by local churches. Lastly, the closest relationship between a Village and other types of associations is that some Villages were established by other associations, including faith-based organizations, senior centers, and the Area Agencies on Aging (AAA).

#### 3.1.5. Governmental Activism (H4)

As there are competing hypotheses about how governmental resources would trigger social entrepreneurship and therefore support the establishment of Villages, the interview findings support Hypothesis 4 that governmental resources are beneficial to Village development. Having lived in the DC metropolitan area for more than 20 years, one interviewed Village director suggested that more governmental activities might be one reason explaining why the number of Villages in the DC metropolitan area is higher than in other places. According to the interviewee's thoughts, although governmental expenditure does not directly lead to social entrepreneurship, people who have frequently participated in governmental activities have the mindset of creating programs providing social services.

…people who come to DC are often brought here because of the government or they're connected to the government in some way as being consultants to the government. People are always thinking about programs and other services, government services, social services, so the people who come here have that in mind. Quotation 6

People in the DC area are often either experienced in relevant affairs or attracted to the area due to their passions for social and public services. In addition, people who have participated in social service programs are more likely to know where to get the resources needed when they are trying to establish Villages. In contrast, while the competing hypothesis suggests that insufficiency in public resources would trigger social entrepreneurship that would spark the establishment of Villages, competing hypothesis does not seem to be supported by the experiences of the Villages' development. The strength of Village services, networking communities and getting to know each individual, is hard for any government program to accomplish. Thus, an insufficiency of standard government services is not needed to spur Village development; the Villages supply the social support that governments seldom provide.

According to the interviewees, even in places with a greater supply of government resources, there are challenges that are hard for governments to overcome when it comes to older adult services. First, many people do not like to deal with governmental staff for various reasons. For undocumented immigrants, they do not want the government to find out where they are even though the government staff does not check their legal status. Some other people are reluctant to look for governmental resources due to the stigma of lazy people relying on social welfare. In addition, many people simply do not know that the government provides the resources they need and also have no intention to search for the relevant information, perhaps because they do not expect the government would provide those resources. The following quotations are provided by a Village coordinator experiencing these issues:

Sometimes when you come from the government, people don't really like you, I get that too…

The county offers a lot of services, but our residents don't always know everything that we do… I don't know why or how that is, I think sometimes people just go about their daily lives and don't think about all of the different opportunities there are in the county. I'm not sure how or why, but so many times I hear, “I never knew you did that.” The county oftentimes offers a lot more than the residents know that we do, particularly our communities that speak a different language. Quotation 7

Furthermore, due to regulatory restrictions and the limitation of governmental actions, even in areas with abundant governmental resources, the government cannot provide certain kinds of services. The government can only provide services to satisfy more basic and general needs; it cannot provide more customized services. For example, while a local government could have a shuttle bus program that carries older adults to the senior centers or supermarkets several times a day, it is difficult for the shuttle to carry older adults to a clinic for a doctor's appointment and take medical notes for the older person it serves. An interviewee also mentioned their Village has members who requested driving services for traveling just a few blocks because those members just had hip or knee surgeries. Moreover, according to a case Village hosted by the AAA, the reason that the agency chose to establish a Village to provide services, instead of providing services directly, is that the Village organization has more capacity and flexibility. In contrast, the AAA has restrictions with regard to state and federal funding.

It's a good partnership; the AAA pays for promotion of the Village, recruitment, mileage for the volunteers, home modifications, small home repairs, utilities, and we [Village] pay a percentage of the Volunteer coordinator's salary. Quotation 8

Nongovernmental organizations such as Villages could address many of the above challenges faced by the government. On one hand, with the strength of tightly networked communities, Villages could actively guide or advise their members to get the governmental resources they need. Compared to the government agencies, they are better at connecting those who need help and those who want to help. Villages are also easier to connect to the people who do not like to interact with governmental staff. In addition, they have fewer legal restrictions than governmental agencies. On the other hand, due to their limited scale and resources, it might be hard for Villages alone to service all those in need. Therefore, rather than being a replacement for governmental resources, Villages are more of a complement. It is less likely that government resources would have a crowding-out effect on the Village development.

**Figure 9:**
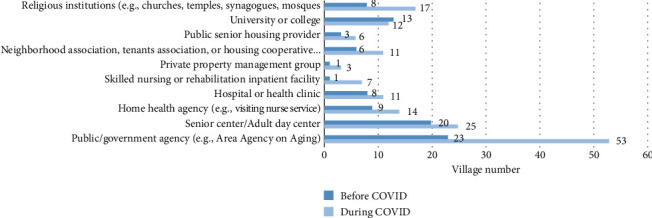
Type of cooperate organization before and during the COVID-19.

During the COVID-19 outbreak, this complementary relationship among the government, other nongovernmental organizations, and Villages was demonstrated. For example, the Arlington Neighborhood Village worked with the AAA, the Arlington Food Assistance Center, and the Arlington Partnership for Affordable Housing to provide services to nonmembers in Arlington County, Virginia. All other three organizations made referrals to the Arlington Village for people looking for help. Those people might be those unable to go to the food center to get food. Arlington Neighborhood Village treated these people as their temporary members. While many residents then were willing to help with its next-door program, Arlington Neighborhood Village could take records on what kinds of help those new volunteers could provide, and the Village also trained those new volunteers so they could better know how to respond to requests from older adults. The survey response also shows some Villages partnering with their city government or police department to help the older nonmembers or homeless. In addition, Figure 9 also demonstrates this complementary relationship between the government and Villages: the number of Villages cooperating with governmental agencies has grown more than 100% after the COVID-19 outbreak; this growth is the highest compared to the growth of partnerships with other types of organizations.

**Figure 10:**
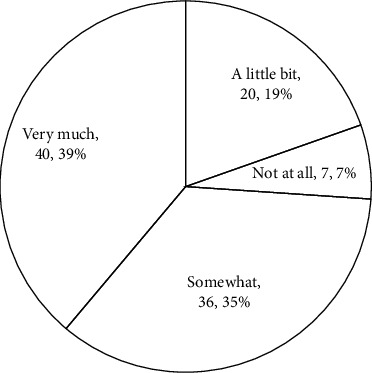
Government Officials (e.g., local or state elected officials, or relevant government agency directors) are supportive of the Village (total participating Villages: 120).

The result of the Village survey also supports this complementary relationship between the government and Villages. Figure 5 indicates many Villages have partnerships with the government. Also, as shown in Figure 10, more than 75% of Villages participating in the survey get at least some of their support from the government. For the areas having more Villages, their local governments are more likely to have resources specifically assigned to Villages, such as having Village coordinators. Also, in these areas, the government officials are more likely to learn about Villages and notice their value. Since the Village concept is new, even in a place with more Villages such as the San Francisco metropolitan area, an interviewed Village manager said that the officials of the local government funding Villages do not fully understand the Village concept. Another fact supporting this mutually reinforcing relationship between government support and Village growth is that, until now, all three of the local governments having Village coordinators to assist Villages are in the DC metropolitan area, the region with the highest in Village numbers and density.

#### 3.1.6. Spatial Proximity and Accessibility (H6)

**Figure 11:**
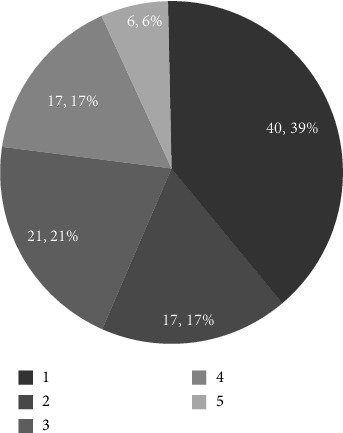
Ease of finding gathering spaces in the Village service area (1 is very easy and 5 is very difficult).

The finding supports Hypothesis 6. Several interviewees mentioned that higher population density and gathering-space availability within the Village service areas would be an advantage for developing Villages. Higher population density makes it easier for people to meet each other physically. In addition, while, as mentioned in Section 3.1.3, the role played by Villages is more equivalent to the faith-based community center in traditional societies, one main difference between the two is that Villages do not have physical gathering spaces as the other one does. Thus, the availability of gathering spaces, such as a community clubhouse, is very helpful to Village development, as one Village coordinator also mentioned. The importance of gathering space is also supported by the fact that the majority of Villages participating in the survey do not have difficulty finding gathering spaces, as shown in Figure 11.

Moreover, spatial proximity improves the spread of the Village concept. If there are Villages in one area, people there have more chances to know or meet someone who participates in or knows about Villages. Two interviewed Village managers had been volunteers for other Villages in the same city before they became managers. Also, since the Village operating models differ to accommodate local situations, it is easier to apply the experiences of nearby Villages than those of faraway Villages. In addition, since the Village concept is new and not easy to be fully understood, the spread of the Village concept relies heavily on word of mouth. This fact also makes spatial proximity important for the Villages' development.

##### 3.1.6.1. Rural Areas

As spatial proximity and accessibility are beneficial for developing Villages, it is understandable that rural areas have fewer Villages. In addition to lesser population density and numbers, the social network is another reason explaining why rural areas have fewer Villages. Compared to other geographic settings, rural areas are more likely to keep the traditional social relationship: family or close friends live nearby, and local churches still play the role of the community center. An interviewee who serves in an older adult services organization in rural areas made a comparison between rural and urban areas as

In a rural community, when they won't have as much support from any government agency because it's hard to reach everybody, what we see is that a lot of the base community of the churches, they will come together to support people or organizational communities, really tremendous support…it's really very different. I have not seen [in urban area] the base community come together where they're able to provide food for people. They reach out to people with telephone calls. They help transportation… Quotation 9

**Figure 12:**
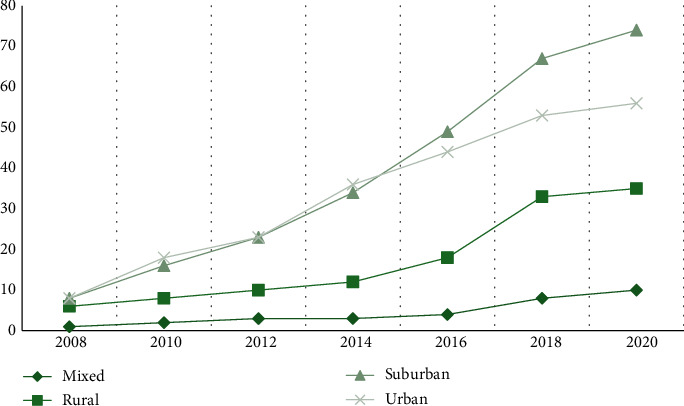
Growth in Village number and geographic setting comparison (total Village number: 197) (Sources: 2020 Village survey response, Village-to-Village Network, Washington Area Villages Exchange (WAVE).

Thus, being networking organizations, Villages are less needed in rural areas. Nevertheless, some rural areas still need Villages and therefore have developed them. The needs and challenges of rural Villages are quite different from those of urban and suburban Villages, including long travel distances and much fewer medical resources. People from a Village in upper New York State even said some of their members and volunteers move to southern areas of the country during wintertime. 9 To address those challenges, some rural Villages have developed unique operating models. While, as mentioned in Section 3.1.3, some Villages in urban and suburban areas only provide social events, there are rural Villages that do not have any social events. It is hard to get people together in rural areas, especially in colder regions. Those rural Villages only provide services such as driving or visiting, and social opportunities occur during the service delivery processes.  Figure 12 also shows that the number of rural Villages has grown since 2014. This fact suggests there is a need for Villages in rural areas.

#### 3.1.7. Income (H7)

The findings support Hypothesis 7, which suggests that the Villages model is more popular in middle- and middle–high-income communities. Establishing a Village requires significant effort, making it less likely that individuals with greater life burdens will start or manage one, particularly in the early years when few examples existed. An interviewee also noted that the needs of lower income communities differ from those of higher income ones, meaning the experiences of middle-class Villages cannot be directly applied to lower income areas without adaptation. The result of the regression analysis also supports this causal relationship. 10

However, case studies show that lower income communities have found alternative ways to establish Villages. All three Villages examined in lower income areas were founded by external organizations, churches, the AAA, and a for-profit corporation. With support from these organizations, including funding, space, and staffing, it becomes more feasible for lower income areas to create their own Villages. Furthermore, social connection is a universal need. Wealth does not guarantee friendships, and even individuals in affluent communities or retirement homes, where services are accessible, may still seek Villages for social engagement. Survey data confirm that some Village members live in retirement communities. While Hypothesis 7 assumes that high-income individuals would have little interest in Villages because they can afford necessary services, this assumption is not entirely accurate. The result of regression analysis further supports this conclusion.

#### 3.1.8. Cultural and Language Barriers

As Figure 5 shows, compared to the United States' average, the percentage of ethnic minorities among the Village members is much lower; this gap might be caused by the nature of Villages along with path dependency. 11 One Village coordinator said that, in a very diverse community, it would be more difficult to get people together to form intimate relationships. People with different backgrounds might not only speak different languages but would also have different subcultures, prefer different food, and enjoy different activities. Therefore, considering the limitation of the Village resources, it might be difficult for a Village to serve members of more than two ethnic backgrounds simultaneously. According to a case Village composed of half Chinese Americans and half non-Hispanic White Americans, the Village needs translators in all its events to ensure the participants understand each other. Furthermore, as suggested by the same Village coordinator, biases, prejudices, and racism also make it hard for a Village to be culturally diverse, although not impossible.

These language and cultural barriers also impede the spread of the Village concept. Since the Village concept itself is already not easy to fully understand, it is harder to introduce the concept to people speaking other languages. Especially, for some ethnic minorities, the older ones are usually first-generation immigrants who do not speak English well. Also, on average, people from different ethnic backgrounds do not hang out together after work as much as people from the same backgrounds. Thus, even though the current White Villagers want to promote the Village concept to ethnic minority communities, they might not be able to find access to do so. Furthermore, like lower income communities, the ethnic minorities cannot directly copy the Village experiences from White communities. It takes time and energy to recreate the Village model to make the revised Village models applicable to the situation in the ethnic minority communities. An interviewee from an ethnic minority also points out another challenge for building a Village of minority: People of the same minority do not necessarily live closely together.

In addition to the above reasons, other facts also make Villages less popular among ethnic minorities. First, some ethnic minorities, especially those who are first-generation immigrants, think it is the family's responsibility to take care of older people, even though, due to the social structure in the United States differing from their home countries, their adult children might not be able to meet their expectations. Second, people of some ethnic minorities do not have the idea of organizing civil organizations to address their needs. This could also explain why the Village concept was born in the United States. As described by Tocqueville [48], Americans have had a very vibrant civil society with all kinds of civil organizations since the very beginning of the country. Tocqueville pointed out that where Europeans seeking support looked first to the government, Americans looked first to form an association for themselves. Third, some newer immigrants might not be aware that social isolation is an issue for older adults. They might live in more traditional societies back in their home countries where most people live in extended families, so they have no chances to see older people suffering from loneliness. They might think affluent material life is enough for their older adults to age well. In fact, even in the United States, according to an interviewee, most people did not identify social isolation as a problem before and during the 1990s. This is another reason why the Village concept was born in the 2000s. Fourth, many ethnic minorities already have organizations that serve similar functions as Villages do. According to an interviewee managing a black church, most African American churches have a ministry that focuses on older people. Also, in the DC metropolitan area, the Chinese Culture and Community Service Center (CCACC) provides older adult services to Chinese Americans in the area, including educational programs, home healthcare, a workshop on dementia, and even a daycare health center. 12

However, although ethnic minorities might have less motivation and more challenges to establish Villages, the Village concept still provides value to them. A black church in the DC district started a Village to serve residents in Ward 8, DC. According to that Village director, compared to providing services directly by the church, establishing a Village to do so produced more concentrated efforts, a wider range of services, and more focus on the older-adult–specific issues such as social isolation. Villages could also provide services to people from different religious backgrounds. For that church, the Village concept provided an opportunity to have more resources to do more. Indeed, all the case Villages containing a higher proportion of ethnic minorities are Villages affiliated with other associations such as churches, senior centers, and private entities. It seems that the Village model could serve as a supplement to the established organizations of ethnic minority communities. Moreover, since interest in participating in Villages very much depends on individual needs and preferences, the general feature discussed in this section would not be applicable to individual situations. An interviewee of Hispanic background also said that it is completely possible for Hispanic people to be interested in Villages. In fact, a Hispanic Village has been established in Florida; the regression result also suggests that Hispanic communities are not less likely to have Villages. 13 Therefore, the Village concept would still have value for people of ethnic minorities. It just takes more time for the Village idea and model to cross the culture and language boundaries and to be reinvented to benefit more people.

#### 3.1.9. Other Factors Contributing to the Village Movement

##### 3.1.9.1. Volunteerism

As the Village model largely depends on volunteers, most interviewed Village volunteers have had many volunteer experiences in the past. In a board meeting of a Village, one person asked, “How many of you were a girl scout or a boy scout growing up?” Everybody raised their hand. Especially for those who have more time due to retirement, Villages provide a good opportunity to give back to the community. One volunteer said, compared to the experiences of volunteering at other nonprofit organizations, Village volunteers have more chances to directly interact and connect to members. Also, since many Villages have member groups for members with common interests, as Quotation 6 on p. 23 mentioned, serving in those groups, such as being a lecturer, also satisfied the need of sharing interests. Thus, volunteerism is definitely a contributor to the development of Villages.  Table 10 suggests that most Village volunteers are residents in Village service areas, and more than 70% of the volunteers are age 60 or more.

##### 3.1.9.2. Motivations Inspired by Older Generations' Experiences

From the very first Village, the aging issues faced by the older generation were an important motivation for people to start Villages. Due to the older generation's experiences, the founders are determined to work on a way to help older people stay at home and not be lonely.

The informal think tank (the first Village founders) had individually experienced issues with their parents' aging. “All were fraught with negativity and based on the concept of ‘move away,'” says Susan. Some involved care facilities and others the trend of selling the family home and moving to a good climate away from loved ones. Susan's mother lived in her own house where she died at 103. “But she was incredibly lonely,” she recalls [49]. Quotation 10

Here is another relevant experience given by an interviewed Village founder who is not a retired person. She witnessed how her older generation suffered from social isolation, and thus saw the value of the Village concept.

I started this Village. I read about it (Village concept) after my mom passed away in 2017. My dad had been taking care of her and actually, we have a lot of family members. We lost a lot of family members and the people who were left, my dad, my aunt, my mother-in-law, they really had a hard time adjusting to being alone. They were still thriving, they were still very involved, but they had a hard time getting back into social activities by themselves. I read about the Village movement and I just really, we're living a lot longer, all of us, we're living longer, and I just felt like we need to do a better job of aging, and I just thought it was a brilliant concept. Quotation 11

Another interviewee was also inspired by her experience of visiting her mother at the assisted living facility. She could not imagine what people did without loved ones to assist or even visit. The above experiences also suggest the value of intergenerational interactions. By interacting with the older generation, the younger people could understand more about the issues faced by the older generation and therefore start to prepare for the future.

##### 3.1.9.3. Balance of Independence and Social Connections

Since many Americans value independence, compared to other possibilities such as being taken care of by children, the Village model provides the option of maintaining a high level of independence when getting older. Simultaneously, as mentioned in Section 3.1.8, from the 2000s social isolation started to be identified as an issue, and people began to notice the importance of networking. According to an interviewee, during the 1980s and 1990s, although the social structure had shifted from extended families into core families, people did not know social support and connections are important for their well-being. This ignorance combined with an individualistic mindset-, not wanting to be burdens for others, made the Village concept less possible before the 2000s. Therefore, this need for a balance of independence and social connections created the Village concept during the 2000s.

##### 3.1.9.4. Social Entrepreneurship

As Section 3.1.5 mentioned, the government-related activities around the DC metropolitan area might attract people who are passionate and good at designing social service programs. While Urbano et al. [50] indicated that social orientation also has positive influences on social entrepreneurship, these people attracted to the DC region can be seen as people with social orientation. 14 Also, as suggested by the manual of the first Village, the Village creators must have “an entrepreneur's willingness to experiment, learn from trial and error, and forge ahead” ([43], p. 4). This suggestion is true for not only the first Village but for all Villages, because every Village needs to be recreated to accommodate its community situation. “We saw an opportunity, and we grabbed it; it was something that sometimes we did not hesitate,” said a manager from a Village implementing new programs during the COVID-19 outbreak and thus doubling its member and volunteer numbers.

##### 3.1.9.5. Membership Fee

Some believe Villages charge high membership fees, making them an unaffordable option for aging in place. 15 However, survey data suggest otherwise. Tables 11 and 12 show the frequency distribution of individual annual membership fees, with an average cost of around $300 per person. Many Villages charge low or no fees at all. Two factors determine how high the Village membership fees are. One is operating costs such as rent for office space. The other is whether the Villages have funding sources other than membership fees such as donations or grants. How many other funding sources a Village could get depends on the availability of funding opportunities and resourcefulness of the Village operators. For example, whether there are nonprofits or governmental agencies providing grant opportunities and whether the people running the Villages have the ability to identify those opportunities and compete for the grants. That is why human capital, such as higher educational levels or relevant working experiences, could help to establish Villages. Village coalitions also host grant writing workshops to help Villagers get more fundraising abilities.

While comparing the cost and services of Villages and other care providers serving similar conditions of the older population, it seems that an average $300 annual membership fee is not that expensive considering the services Villages provide. Moreover, according to the field observations, a Village member, who lives in a single-house community in Fairfax County, Virginia, said that the driving service provided by her Village saves her from spending on Uber, and it is one of the main reasons for her to join the Village. 16 In fact, among its members, the driving service is the most popular service provided by that Village. This example shows that even for people living in higher income communities, saving money could be a reason for them to join Villages. This example also suggests that, when judging whether the Village membership fees are expensive, one needs to compare the Village fees with the costs of the alternative ways to get similar services rather than solely look at the amount of the Village fee. In addition, many Villages also provide multiple types of membership so the members could pay a lower cost if they need fewer services. However, as mentioned in Section 3.1.7, for lower income areas, it is difficult to have a Village operating solely on membership fees. Thus, for communities of lower income, it is more important for the Village builders to find partnerships or sponsorships.

#### 3.1.10. Special Operating Models of Villages (Q2, Q3)

As shown in Table 3, in addition to typical standalone Villages, the Village model has other subtypes. Since different models accommodate different community situations and help to sustain Villages, describing and discussing those models will help us answer the second and third research questions.

##### 3.1.10.1. Hub-and-Spoke Villages

**Figure 13:**
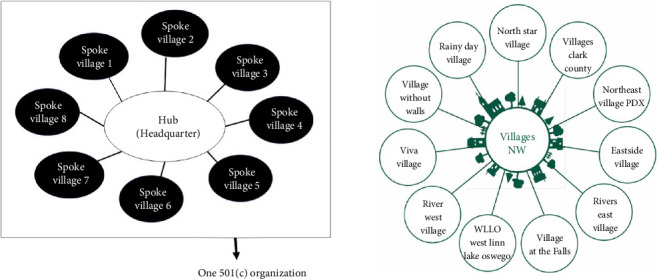
Hub-and-spoke Village model and the example of Villages NW (source: Villages NW [51]).

**Figure 14:**
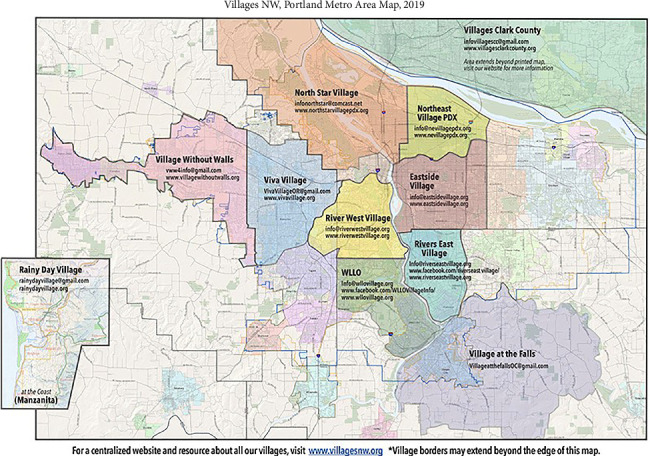
Service areas of the spoke Villages of Villages NW [52].

Briefly speaking, the model of hub-and-spoke Villages is one Village having branches serving different areas, as Figures 13 and 14 show. The hub carries out all the office paperwork such as banking, insurance procurement, and legal procedures. It does not serve any members directly. Instead, the hub serves the spoke Villages, so that the latter can focus on recruiting and serving members and volunteers. In turn, the spoke Villages share the operating cost of the hub. Compared to the standalone Villages, the hub-and-spoke model significantly lowers the cost of starting a Village. For the spoke Villages, they only need to have their leadership teams and to be financially independent. Also, with all the spoke Villages and their service areas, it is much easier to get funding opportunities.

The Villages NW is the first Village applying this model. In 2011, the Village founder launched a website to attract and bring together people who would be interested in developing a Village on Portland's east side. After talking to people from across the metropolitan area who wanted to start a Village in their neighborhood, the founder realized that the areas with people wanting to have a Village were too large to be served by one Village. After consulting with the global Village coalition, she found a way to develop Villages across the Portland metropolitan area while sharing resources among those Villages. That is, the hub-and-spoke model developed by Villages [53]. Their experience shows that the hub-and-spoke model is suitable for establishing Villages to serve larger areas using a common hub. Compared to the Village coalitions mentioned in Section 3.1.4.1, the hub-and-spoke model is a more intimate way for Villages to share resources.

One might want to ask: how many spoke Villages can one hub serve effectively? The answer to this question lies in the pros and cons of the model. While the hub-and-spoke model could reduce the founding cost per Village, an increase in communication cost occurs due to joint decision-making among all the spoke Villages. According to the experience of Villages NW, policy decisions of the board are taken to a circle of representatives including all 11 spoke Villages for recommendations, and voted on by all the spoke Villages. This can be cumbersome if the Villages do not have much in common. Thus, to make the model beneficial for a Village, the amount of reduced founding cost should be larger than any increased communication cost. Therefore, the spoke Villages served by one hub should have some similarities, such as being geographically not too distant from each other so they share the same regional situation. The number of the spoke Villages also should not be too high. According to Villages NW's experience and the 13 Villages in the DC district applying and managing grants jointly, the upper bound of this number might be 15.

##### 3.1.10.2. Villages Developed by Other Organizations

While Section 3.1.4.2 shows how Villages have obtained support from other organizations during their founding and operating process, some organizations even start Villages on their own. These organizations include churches, the AAA, senior centers, homecare nonprofit organizations, and a private corporation wanting to do charity. Village foundations could either be initiated by the host organizations or by the residents wanting Villages. As for legal status, some of those Villages are affiliated with their host organizations. Some of them are registered as independent nonprofit organizations after providing services for a while. Some host organizations even have more than one Village affiliated with them.

For these organizations, Villages serve as a supplement or extension for helping achieve the purpose of their organizations. By establishing a Village, an AAA official said the agency had more capacity and flexibility in providing services, as shown in Quotation 8 and the paragraph before the quotation. By establishing a Village, a church already having some older adult services can have more concentrated efforts, more resources, a wider range of services, and the ability to serve people with different religious backgrounds, as noted in the last paragraph of Section 3.1.8. By establishing a Village, a senior center can reach out to communities rather than just stay inside the senior center; this benefit was particularly valuable during the pandemic outbreak. By establishing a Village, a for-profit company running a lower income older adult community can address the social isolation issue in the neighborhood. By establishing a Village, a homecare nonprofit organization can be more capable of achieving its goal of keeping older people and the disabled at home. 17

The pros of this affiliated Village model are that with the resources from the host organizations, including funds, social networks, physical spaces, and human resources, the cost of starting a Village can become much lower. Also, by being affiliated to an established organization, the Village founders and operators could avoid the cost of the legal procedures for registering to be a nonprofit organization and submitting required documents to the government annually. These two advantages are particularly helpful for communities with fewer income and resources. Being alone, those communities might not be able to establish their own Villages. The following opinion was provided by an interviewee who thinks that the models such as the Villages or naturally occurring retirement communities (NORCs) are a good way for ethnic or religious groups to provide services for older adults.

Supporting neighbors, especially older adults, is an important value of many ethnic and religious groups. Because these groups often have established trusted relationships with people of all ages in their community, there is more buy-in and comfort for individuals seeking services with the businesses, organizations, and volunteers who provide assistance. It's important to note that our NORC engages and serves people of all faiths and backgrounds, just like many of our community organizations do (e.g., the Jewish Community Center). Quotation 12

While the benefits are obvious, this model also has drawbacks. Unless the affiliated Villages are registered as independent organizations later, the activities of this kind of Village would be restricted by the agenda and rules of the host organizations. Moreover, if the managers of Villages and host organizations are not the same people, Villages also have the cost of communicating with the host organizations. A case Village started by a church that became independent after 3 years of the operation, said by becoming an independent nonprofit organization, the case Village was able to reach out to more funding opportunities.

As the key feature of the Village concept is the grassroots' approach to help people aging in place, is it still a Village if a Village is started not by community residents but by an established organization? Indeed, as long as Villages attract high participation by local older adults and are able to network with communities, it really does not matter who built the Villages. Moreover, several affiliated case Villages explicitly acknowledged that they were inspired by and learned from other Villages in the interview.

##### 3.1.10.3. NORCs

The data show that the distinctions between Villages and NORCs are obscure, if any. This research found that, indeed, both Villages and NORCs have diversity. Both Villages and NORCs could range in size from a building, a neighborhood, to a county, and incorporate the services provided by professional social service providers. Both could be operated by a private entity or a public–private partnership and be standalone nonprofit organizations or affiliated with other organizations. The only thing making one retirement community a Village or NORC is which example inspired the organizational founder. While so far, this research has already mentioned several Village founders who were inspired by the Village concept from other Villages, the founding of the two NORC cases of this research was inspired by other existing NORCs. Perhaps the major difference between Villages and NORCs is that, unlike Villages, NORCs do not have active coalitions as Villages do. According to a NORC interviewee, there used to be a national network of NORCs, but it has not been active in at least the last 5 years. That is why, in another case, NORC chose to join the Village global coalition for networking and educational purposes.

### 3.2. Regression Analysis Results

#### 3.2.1. Model Selection and Regression Result

While ZINB and ZIP models are chosen to carry out the analysis, according to the value of log-likelihood shown in Table 6, the ZINB model is better to analyze the data because its log--likelihood values are smaller at both county and county–subdivision levels. Moreover, shown in Table 6, because the results of Vuong test are significant, compared to the negative binomial model and Poisson model, the ZINB model and ZIP model are better fits. At the county–subdivision level, standard Poisson and negative binomial models failed to converge, further supporting the choice of zero-inflated models.  Table 13 is the regression result of ZINB regression analysis at both county and county–subdivision levels with open and total Village numbers as dependent variables. 18 In the table, each variable explicitly indicates which hypothesis it tests.

#### 3.2.2. Explanation and Discussion of the Regression Result

The explanation of the regression results is based on two principles. First, if the results at the county level and county–subdivision level differ, the latter is considered more reliable. This is because the scale of the county subdivision more closely aligns with the Village service area. Second, compared to the model using open Village numbers as the dependent variable, the model using all Village numbers provides insight into future trends, as it includes Villages in development. The following discussion categorizes explanations and interpretations by independent variables and research hypotheses, with a concluding summary at the end.

##### 3.2.2.1. Percentage of Population Aged Above 65 and 85 (H1)

The analysis confirms that areas with a higher percentage of residents aged 65 and above are more likely to develop Villages. This supports the hypothesis that demographic shifts in aging populations contribute to Village formation. However, while the percentage of residents aged 85 and above does not consistently show significance, the county–subdivision level results indicate a growing trend of Village establishments in areas with a higher proportion of this age group. This suggests that as populations continue to age, Villages may become increasingly relevant in supporting older adults.

##### 3.2.2.2. Cost of Care: Prices and 5-Year Price Growths on the Homemaker Services, ADHC, and Assistant Living in Semiprivate and Private Room (H1)

While the rising care cost is expected to be a factor of the Village movement, the regression results have some inconsistency with the hypothesis expectations. While the services of homemakers and ADHC are considered to be similar to the Village service compared to that of assistant living, some of the regression results are either insignificant or contrary to the hypothesis expectations. This inconsistency suggests that, although providing some home services, the Village services could not be taken as a replacement for the care provided by homemakers or ADHC. The value for the Village service is more about keeping older adults socially and physically active in the communities. For example, the Village services such as driving or educational programs are not provided by homemaker services.

##### 3.2.2.3. Human Capital: Percentage of People With Bachelor's' Degree or Higher and Percentage of People With More Management-Related Jobs (H2)

The positively significant results indicate that higher educational attainment and management-related work experience positively correlate with Village formation. This aligns with Hypothesis 2 that communities with more human capital are better positioned to develop and sustain Villages. In addition, the analysis result shows that the role of management experience may be particularly influential, as it provides organizational and resource-mobilization skills essential for launching and maintaining Villages. However, the management-related job variable might suffer from a measurement bias: other occupation categories, such as education, might also include management-related positions. Despite this measurement defect, these positively significant results confirm the finding of the Village case study: older adults with more human capital would be more interested in participating in Villages and also more capable of getting resources for operating Villages. 19

##### 3.2.2.4. Social Capital Index and Religious Organization Percentage (H3)

The regression analysis gave a robust result on the impact of social networks: the value of the social capital index is positively associated with the Village count, while its square value is negatively associated with the Village count. Whereas this research expected that both well-connected and fragmented community social networks could give an advantage or incentive in starting Villages, the regression along with the interview data found the relationship between the social networks and Village establishment is indeed concave: moderate levels of social connectivity facilitate Village development. In contrast, very strong social networks may reduce the need for Villages. In addition to the social capital, the result of the religious organization percentage variable also confirms the findings of the Village case study: While the case study suggested that the role that Villages play is similar to the faith-based community center in a traditional society, the negative significance of the religious organization percentage variable also suggests the substitute relationship between Villages and religious organizations.

##### 3.2.2.5. Governmental Expenditure/GDP (H4)

While Stephan et al. [23] indicate two opposite impacts of governmental activism on social entrepreneurship, the positive significance result shows that, in the case of Villages, governmental activism does indeed trigger social entrepreneurship. The qualitative study performed by this research also suggests that Village services are more complementary than substitutive to the services provided by the government. These findings support Hypothesis 4.

##### 3.2.2.6. Regional Culture Encouraging or Discouraging Participation and Cooperation (H5)

**Figure 15:**
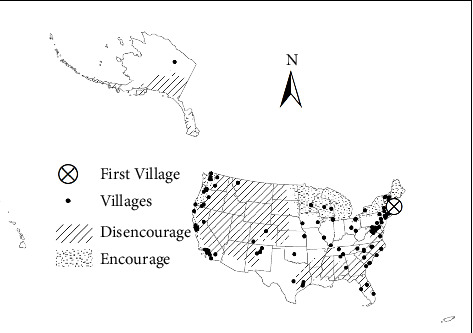
Village distribution in the areas with the regional cultures that encourage/discourage participation and cooperation30.

At the county–subdivision level, areas with a regional culture encouraging participation and cooperation have a positive association with the Village count. This significant result is consistent with Hypothesis 5. The result also suggests that this culture could be beneficial in establishing a Village. In fact, the birthplace of the first Village is also located in a place with the encouraging regional culture. In contrast, regional culture discouraging participation and cooperation does not have any significant impact on the number of Villages. This result could be explained by the fact that the regional culture identified by Woodard [24] is based on historical context. Nowadays, those historical impacts are largely diluted by industrialization and information technology, especially in more urbanized areas. Thus, this discouraging culture does not affect the Village movement.  Figure 15 shows the distribution of Village locations in the United States along with the areas with regional cultures that encourage or discourage participation and cooperation.

##### 3.2.2.7. Spatial Proximity and Accessibility (H6)

Although this research failed to use the spatial regression model to carry out the analyses, the results of the spatial effect variable show robust positive significances. This result supports Hypothesis 6 and the findings from the Village case studies: Villages in nearby areas could be helpful in experience learning when people are trying to start a Village. Moreover, since this study assumed that a high density of population and roads would increase the opportunities for social interactions, it was expected that both density variables would be positively associated with the Village count. However, the result of population density is insignificant; moreover, the road density result is even negatively significant at the level of 0.1. These results suggest that population and road density are not very relevant to the Village establishment. Particularly, considering that the United States has a generally functional transportation system, the road density does not make too much difference for people to commute to nearby areas.

##### 3.2.2.8. Income (H7)

The result is generally consistent with the expectation. It does suggest that higher income areas are more likely to build Villages. Yet, at the county–subdivision level, the square of household income is not significant. It suggests that very high income might not have negative impacts on the Village establishment. The qualitative study of this research points out that some benefits provided by Villages, such as social connections, cannot be obtained through paid services. Therefore, people with very high incomes might still want to participate in a Village.

##### 3.2.2.9. Percentage of Charitable Donation Over Income

The insignificance of this variable suggests that charitable donation does not have an identifiable association with establishing Villages. This result might suggest that starting a Village does not have too much to do with people's willingness to make monetary donations. Another explanation for this insignificance is that many charitable amounts might be contributed for tax avoidance purposes. Thus, charitable amounts percentage might not be able to represent volunteerism or willingness to help.

##### 3.2.2.10. Ethnicity Percentage

While the survey data suggest that Villages might be more likely to exist in White communities, the regression result suggests this might not be true after controlling all other factors such as income and human capital. 20 (Table 14) This result suggests that lower income, educational level, or younger population, rather than culture or ethnicity, might be the reasons that some minorities have fewer Villages. In addition, the weak negative significance at the county–subdivision level of the White population percentage variable could be explained by the fact that, compared to other ethnic groups, White people might be more likely to live in areas with stronger traditional social networks, such as more rural areas. Moreover, the presence of these traditional areas could not be identified by population density only since the population density value is not significant.

The regression result also shows that a higher Asian population percentage is negatively associated with the Village count. Here is the explanation: The Village case study result suggests it would be easier to establish a Village when the community members have some homogeneity. Therefore, Asian communities are more difficult to start Villages because, compared to other ethnic groups, they have not only fewer populations but also larger cultural and linguistic diversity. Moreover, the case study result suggests that the Asian Village members are more likely to be Chinese or Japanese probably due to the larger population of the ethnic groups. Yet, the exact reasons for this phenomenon need further study.

## 4. Conclusions

Building upon the findings from the survey and case studies, it is evident that the Village model is not merely a provider of services but a mechanism for rebuilding and sustaining community social networks. The results indicate that while some communities naturally possess strong social ties, others require intentional efforts to recreate these networks. The Village model serves this purpose by fostering engagement and participation among older adults, reinforcing social capital and offering opportunities for meaningful involvement. This aligns with Maslow's hierarchy of needs, which suggests that beyond basic needs, individuals seek social belonging and self-fulfillment [54]. The emergence of Villages can be attributed to shifts in societal structures and attitudes toward aging. Unlike previous generations, older adults today are more aware of the importance of social connections and are proactive in seeking community engagement. While various models of Villages exist, their core function remains the same: to restore and strengthen local social networks. This function differentiates Villages from traditional service providers and informal social networks, which may offer similar support but lack the foundational goal of network restoration.

This uniqueness of Villages is not only indicated by the interviewees but also supported by two regression findings: The concave-shaped relationship between social networks and Village establishment suggests that communities with very strong pre-existing networks may lack incentives to develop Villages. In addition, little correlation between the number of Villages and the availability of traditional older adult services, such as home care, underscores the unique role of Villages in addressing the nonphysical aspects of aging. The fact that older adults who can afford care services still choose to participate in Villages indicates that Villages provide an essential social function that complements but does not replace existing care infrastructure.

Furthermore, regression analysis and case studies reveal that several factors influence Village formation and sustainability. Yet, none of these factors are absolute, and many of the factors can be developed or overcome by sharing experiences or working with other organizations. Among those factors, higher educational attainment and management-related experience enhance both the motivation and capability to establish Villages. Affluent communities are also more likely to develop Villages due to available resources and stronger motivation. Lower income communities often rely on partnerships with external organizations. This fact showcases the adaptability of the Village model and its potential for broader implementation through institutional collaboration. In addition, governmental activism also plays a crucial role in Village sustainability. Findings suggest that strong governmental engagement complements Village activities rather than substitutes for them. Governmental activism could also attract people with social orientations and therefore increase social entrepreneurship in the areas [50]. Furthermore, both regression and the Village case study show that spatial proximity also significantly influences Village expansion. Communities with existing Villages are more likely to develop new ones due to increased exposure and knowledge-sharing. In addition, the interview and survey data suggest that access to communal gathering spaces helps Village development by facilitating interactions and engagement. These findings align with Durkheim's (1893) theory of social cohesion, which emphasizes the importance of shared spaces in fostering collective solidarity.

The flexibility of Villages as well as their ability to work with other organizations are the key for Villages to become sustainable. For example, affiliating with existing organizations is one way to establish Villages when resources are limited. This model is especially common in lower income or ethnic minority communities. In particular, while the role played by Village is similar to the faith-based community center in the traditional society, this research found that religious groups still provide great value in providing care for older adults. Religious groups also host some Villages or have partnerships with Villages. The number of partnerships between Villages and religious groups had grown twice during the pandemic, as shown in Figures 5 and 9. These findings on religious groups align with existing literature, which highlights their role in enhancing the diversity of community organizations and shaping shared values [55–57]. The literature also suggests that faith-based organizations may engage in dialog and collaboration with other community or social organizations. In addition, learning experiences from other Villages is crucial for Villages to grow and take on the challenges they have.

Since the percentage of the older ethnic minority population is expected to increase in the future, it is important to know whether the Village model would help ethnic minorities. The research findings suggest several reasons for minorities' less participation in Villages. First, members of a Village tend to be homogeneous. It is not only because people like to hang out with people similar to themselves but also because serving an additional ethnic group will lead to a higher and unaffordable operational cost due to offering different types of activities, food, and hiring interpreters. Some ethnic minorities might have a very small population located in nearby neighborhoods, so they have difficulties establishing a Village that mostly serve people of their ethnic group. Second, some minorities on average have lower income, lower educational level, or younger population. As shown in the regression, after controlling income, education level, and age, black and Hispanic population percentages become either positively associated with the Village number or insignificant. This result suggests that it is income, educational level, or age, rather than culture, that causes these minorities to have fewer Villages. Third, some minorities do not have the practice of organizing civil groups to address their common challenges. Finally, some ethnic minorities already have organizations providing functions similar to Villages. Yet, the Village case study shows that Villages in communities of ethnic minorities are more likely to partner with other organizations, such as churches or ethnic groups. Those findings suggest that the Village concept could still be helpful to ethnic minorities.

### 4.1. Relationship Between Villages and Local Governments

As this study found that the services provided by Villages and the government are more complementary rather than substitute, Table 15 identifies and categorizes all kinds of interaction between the local government and Villages from the research data. That interaction with a level greater than three means the governments give more substantial resources in supporting the Village operations, including funds, physical spaces, or staff.

### 4.2. Contribution and Policy Suggestions

This study represents the first in-depth investigation of the Village model since the establishment of the first Village in 2002. Unlike previous research that primarily relied on self-reported survey data, this study employed a mixed-methods approach, with findings from different methods reinforcing one another. In addition to self-reported data from Village participants, the study incorporated third-party sources, including field observations, interviews with government officials and other older adult service providers, and socioeconomic variables used in the regression analysis. Moreover, although COVID-19 delayed survey and interview responses and may have lowered the response rate, it also gave this study an opportunity to observe how Villages responded to an external shock.

The research finding suggests Villages are a good way to reconnect social networks at the community level and therefore to help residents to get more social support. This function of the Village model is particularly valuable in more urbanized areas where people are losing the social networks in their neighborhoods. The research also breaks some stereotypes or myths that people have about Villages. Many people, including those who work in relevant professions, think the Village model only works in communities located in more urban areas and having higher income residents. Also, many people think people of ethnic minorities do not need Villages because they think caring for older adults is the family's responsibility. This research found that the Village model could be adjusted to fit the different needs and conditions of various communities. For example, a Village model developed in rural areas only provides services of driving and friendly visiting. In addition, the expectation that younger family members should care for older adults is rooted in traditional societies, not specific to any particular ethnicity or culture. In more modernized or industrialized societies such as the United States, this expectation is less common. The Village model can still serve ethnic minority communities, and more Villages catering to these groups are being established.

Based on the findings of this study, several policy recommendations can be made to support the growth and sustainability of the Village model as a community-based solution for aging in place.

#### 4.2.1. Support for Village Startups

Villages require the most assistance during their founding stages. Policymakers should consider establishing programs that offer seed funding, temporary office space, and administrative support for emerging Villages. In addition, creating platforms that connect individuals or groups interested in starting a Village with existing resources or mentors, such as regional coalitions or established Villages, could significantly reduce barriers to entry.

#### 4.2.2. Promote Public Awareness

Despite the value of the Village model, public understanding remains limited. Government agencies, especially those focused on aging or public health, should launch targeted campaigns to promote awareness of the Village concept. These efforts should also highlight the diversity of operating models (e.g., standalone, hub-and-spoke, and affiliated Villages) to demonstrate the flexibility and adaptability of the Village approach.

#### 4.2.3. Integrate Villages Into Broader Aging Policy Frameworks

Villages should be recognized and supported as a component of community-based aging strategies. For example, AAA could incorporate Villages into their service delivery networks or provide technical assistance to communities interested in starting Villages. Policy frameworks at the federal, state, and local levels should include provisions to foster collaborations between Villages and formal service providers.

#### 4.2.4. Address Cultural and Language Barriers

To increase inclusivity, policies should promote the development of culturally adapted Village models. This includes funding bilingual outreach materials and supporting partnerships between Villages and trusted institutions in minority communities, such as churches, cultural centers, and ethnic service providers. These partnerships can help overcome trust issues and facilitate the transfer of the Village concept across cultural and language boundaries.

#### 4.2.5. Encourage Diverse and Sustainable Funding Models

To ensure long-term sustainability, Villages need to diversify their funding sources beyond membership fees. Policymakers can help by offering grant opportunities and promoting partnerships with local businesses, foundations, and philanthropic institutions.

#### 4.2.6. Leverage Villages as Community Partners in Public Service Delivery

Given their trusted relationships and close ties to the community, Villages are well-positioned to support local service delivery efforts, especially in times of crisis. Policymakers should consider formally recognizing Villages as partners in emergency preparedness, public health outreach, and care coordination, particularly for hard-to-reach populations.

### 4.3. Limitation and Future Research

Even though this study tried to carry out the research as thoroughly as possible, it still suffers from several limitations and therefore needs future research. First of all, due to the restrictions caused by the COVID-19 pandemic, most interviews and field observations were on management and volunteers. More research is needed with a focus on the Village members or community residents. Furthermore, while this research only focuses on Villages in the United States, it would be valuable to study the experiences of the Villages in other countries.

## Figures and Tables

**Table 1:** Research questions and the corresponding research hypotheses and method.

Research questions	Research hypotheses	Research methods
Q1	H1, H3, H8, H9	Case study, regression
Q2	H2, H3, H4, H5,H6,H7	Case study, regression
Q3	H8, H9	Case study

**Table 2:** Case villages (by research hypothesis).

	Urban/Suburban (H7)	Rural (H7)
Villages in areas with lower income or educational level (H2, H7)	Kingdom Care Senior Village	Klamath Village and Outback Strong Village
Villages of which 30%–95% members are ethnic minorities (H2, H3, H7)	Care Connections Network, Golden Gate Village, Hotel Oakland Village, Kingdom Care Senior Village	(No Village fits this category)
Villages in areas with regional culture discouraging participation and cooperation (H5)	Monte Sano Village	My Glacier Village, Klamath Village and Outback Strong Village
Controlling case	Lake Barcroft Village, Dupont Circle Village, Foggy Bottom West End Village	Rapp At Home

*Note:* The case villages themselves identify whether they are serving low-income neighborhoods or rural areas.

**Table 3:** Case Villages (by establishing approach).

Village type (by establishing approach)		Village cases
Independent villages	Standalone villages	Beacon Hill Village (first Villages), Monte Sano Village, Rapp At Home, My Glacier Village (only Village in the locating state), Lake Barcroft Village, Dupont Circle Village, Wellington Cares (NORCs)
	Hub-and-spoke villages	NW Villages

Villages host/developed by other organizations	Host/developed by church/faith-based groups	Kingdom Care Senior Village, Care Connections Network, St. Louis NORC (NORCs)
	Host by area agency on aging	Klamath Village and Outback Strong Village
	Host by senior center	Golden Gate Village
	Host by other organizations (NPO, for-profit company)	Hotel Oakland Village, JP@Home

**Table 4:** Village case study data sources.

Data types	Data sources	Number of documents (73 in Total)
Interviews: transcript, summary	Case village founders, management (also the Village members), staffs from the 16 case Villages	32 interviews, 10 summaries
	Case Village volunteers: 7 volunteers from 5 Villages	
	A Village coalition board member	
	Three Village coordinators: Montgomery County, MD, Rockville City, MD, Fairfax County, VA	

Others	Case Village websites	9
	Case Village monthly/quarter newsletters, publications	14
	Qualitative responses to the Village survey	1
	Village conferences (2017–2021, national conference and DC metro area conferences): field notes, discussion transcript, presentation slides	6
	Field notes from Village events and a Village board meeting	3

**Table 5:** Observations/analysis units.

Analysis unit	Number	Year21	Selecting reasons	Data source
County/city	3233	2010	The county is the smallest governmental administrative area for implementing aging-related policy.	US census22
County subdivision	35,649	2013	Since, according to the data from the interviews and survey, most villages serve 3–8 zip code areas, the county subdivision is the closest geographic unit defined by the US government. Also, while the US Census Bureau only provides the shapefile of county subdivision by state, the 2013 shapefile of the entire US is available on the ESRI website.	US census, ESRI23

**Table 6:** Model Selection: Vuong test and log-likelihood (dependent variable: open Village number).

	Zero-inflated negative binomial model		Zero-inflated Poisson model	
	Log-likelihood	Vuong test	Log-likelihood	Vuong test
County	−489.0263	2.84^∗∗∗^	−493.9381	3.26^∗∗∗^
County subdivision	−992.9781	5.09^∗∗∗^	−995.5708	5.93^∗∗∗^

^∗^ *p* < 0.1.

^∗∗^ *p* < 0.05.

^∗∗∗^ *p* < 0.01.

**Table 7:** List of independent variables24.

Category	Variables	Unit	Tested hypothesis	Expected direction	Sources	Scale, year		
						State level	County level	County–subdivision level
Population	Percentage of the population aged above 65	%	H1	+	American Community Survey	—	2010	2013
	Percentage of the population aged above 85	%	H1	+				

Care cost	Homemaker services	Dollars	H1	+	“2018 Cost of Care” (n.d.)	2018	—	—
	Homemaker services 5-year annual growth	%	H1	+				
	Adult day health care (ADHC)	Dollars	H1	+				
	Adult day health care 5-year annual growth	%	H1	+				
	Assistant living semiprivate room	Dollars	H1	+				
	Assistant living semiprivate room 5-year annual growth	%	H1	+				
	Assistant living private room	Dollars	H1	+				
	Assistant living private room 5-year annual growth	%	H1	+				

Human capital	Percentage of people with bachelor's degree or higher	%	H2	+	American Community Survey	—	2010	2013
	Percentage of people with more management-related jobs25	%	H2	+				

Social networks	Social capital index26	−3.18 to 21.81	H3	+	Rupasingha et al. [39]	—	2014	—
	(Social capital index + 4)^2^		H3	−				
	Religious organization number/total civic organization number	%	H3	−				

Governmental activism	Governmental expenditure/GDP	%	H4	+/−	US Census Bureau	2019	—	—

Culture	Regional culture encouraging participation and cooperation	Dummy	H5	+	Woodard [24]27	2012	—	—
	Regional culture discouraging participation and cooperation	Dummy	H5	−				

Volunteerism	Charitable donation/income	%	H5	+	IRS	2018	—	—

Spatial effects	Number of neighboring counties with Villages28		H6	+		—	2020	2020

Spatial proximity	Road density	Miles/square kilometer	H6	+	Bureau of Transportation Statistics, US29	—	2014	2014
	Population density	People/square mile	H6	+	American Community Survey	—	2010	2013
Income	Income per capita	Dollars	H7	+			2010	—
	(Income per capita)^2^	Dollars	H7	−			2010	—
	Mean household income	Dollars	H7	+			—	2013
	(Mean household income)^2^	Dollars	H7	−			—	2013
Ethnicity	Asia population percentage	%	Controlling variable	None			2010	2013
	Hispanic population percentage	%	Controlling variable	None			2010	—
	White people percentage	%	Controlling variable	None			2010	2013
Population	Total population	People	Controlling variable	None			2010	2013

Regional dummy	West	Dummy	Controlling variable	None	US Census Bureau	Not applicable		
	South	Dummy	Controlling variable	None				
	Northeast	Dummy	Controlling variable	None				

**Table 8:** Independent variable selections.

Level	Correlation variables		Correlation parameters
	Selected variable	Unselected variable	
County	Income per capita	Mean household income	0.9286
County/county subdivision	Non-Hispanic White percentage	Black percentage	−0.8121 (county), −0.8131 (county subdivision)
County/county subdivision	Home service cost	Home health aide cost	0.9805
County/county subdivision	Assistant living semiprivate room cost	Nursing home private room cost	0.9858

**Table 9:** Village operating status in the US.

Open	317
In development	43
Closed due to COVID-19	4
Participating in this survey	120
Total	364

**Table 10:** Volunteer sources (total participating Villages: 95).

Volunteer sources	Average percentage
Village members	35.22%
Residents in service areas who are not village members	46.77%
Below age 60	22.00%
High school students	2.19%
College students	1.96% (with 19.56% of them being not residents in the service areas)
Not residents in the service areas	2.85%

**Table 11:** Statistics of annul individual membership rate.

Mean	Median	Standard deviation	Max	Min	Number of villages
$293.34	$300	$223.84	$1020	$0	195

**Table 12:** Frequency table of annual Village individual membership rate.

Annul membership rate	Village number	Percentage (%)
$0	23	11.79
$600	14	7.18
$300	13	6.67
$400	10	5.13
Total	195	100

**Table 13:** Regression result.

Geographic scale		County		County subdivision	
Independent variable, tested hypothesis	Expected direction	All Villages	Open Villages	All Villages	Open Villages
Negative binominal part					
Intercept	None	−1.611 (2.713)	−0.762 (2.833)	1.305 (2.447)	1.761 (2.581)
Percentage of population age above 65 (H1, H6)	+	0.121 (0.031)^∗∗∗^	0.119 (0.034)^∗∗∗^	0.032 (0.013)^∗∗^	0.03 (0.015)^∗^
Percentage of population age above 85 (H1, H6)	+	0.142 (0.173)	0.078 (0.183)	0.109 (0.049)^∗∗^	0.096 (0.059)
Homemaker services (H1)	+	< 0.001 (< 0.001)	−< 0.001 (< 0.001)	−< 0.001 (< 0.001)^∗^	−< 0.001 (< 0.001)^∗^
Homemaker services 5-year annual growth (H1)	+	13.564 (10.961)	19.022 (11.615)	9.652 (9.159)	12.999 (9.757)
Adult day health care (H1)	+	−< 0.001 (< 0.001)^∗∗∗^	−< 0.001 (< 0.0011)^∗∗∗^	−< 0.001 (< 0.001)^∗∗∗^	−< 0.001 (< 0.001)^∗∗^
Adult day health care 5-year annual growth (H1)	+	6.117 (3.835)	4.641 (4.023)	4.211 (3.555)	3.186 (3.793)
Assistant living semiprivate room (H1)	+	< 0.001 (< 0.001)^∗^	< 0.001 (< 0.001)	< 0.001 (< 0.001)^∗∗^	< 0.001 (< 0.001)
Assistant living semiprivate room 5-year annual growth (H1)	+	2.328 (8.018)	0.775 (8.504)	−5.19 (7.22)	−5.6 (7.675)
Assistant living private room (H1)	+	< 0.001 (< 0.001)	< 0.001 (< 0.001)	< 0.001 (< 0.001)^∗∗∗^	< 0.001 (< 0.001)^∗∗^
Assistant living private room 5-year annual growth (H1)	+	8.814 (5.724)	8.996 (6.013)	8.348 (4.703)^∗^	8.882 (5.03)^∗^
Percentage of people with bachelor's degree or higher (H2)	+	0.107 (0.016)^∗∗∗^	0.102 (0.017)^∗∗∗^	−< 0.001 (< 0.001)	−< 0.001 (< 0.001)
Percentage of people with more management-related jobs (H2)	+	0.158 (0.044)^∗∗∗^	0.176 (0.045)^∗∗∗^	0.124 (0.026)^∗∗∗^	0.123 (0.028)^∗∗∗^
Social capital index (H3)	+	2.946 (0.84)^∗∗∗^	3.065 (0.879)^∗∗∗^	4.045 (0.784)^∗∗∗^	3.858 (0.824)^∗∗∗^
(Social capital index + 4)^2^ (H3)	−	−0.349 (0.098)^∗∗∗^	−0.351 (0.102)^∗∗∗^	−0.444 (0.094)^∗∗∗^	−0.416 (0.098)^∗∗∗^
Religious organization number/total civic organization number	−	−0.073 (0.026)^∗∗∗^	−0.074 (0.027)^∗∗∗^	−0.035 (0.02)^∗^	−0.038 (0.022)^∗^
Governmental expenditure/GDP (H4)	+/−	10.776 (3.742)^∗∗∗^	10.83 (3.889)^∗∗∗^	9.81 (3.232)^∗∗∗^	9.884 (3.399)^∗∗∗^
Regional culture encouraging participation and cooperation (H5)	+	−0.05 (0.237)	−0.089 (0.246)	0.447 (0.192)^∗∗^	0.381 (0.203)^∗^
Regional culture discouraging participation and cooperation (H5)	−	−0.077 (0.225)	−0.079 (0.236)	0.114 (0.197)	0.161 (0.207)
Charitable amount/income (H5)	+	−0.059 (0.154)	−0.057 (0.156)	0.13 (0.115)	0.098 (0.123)
Number of neighboring counties having Villages (H6)	+	0.3 (0.065)^∗∗∗^	0.282 (0.067)^∗∗∗^	0.274 (0.067)^∗∗∗^	0.271 (0.07)^∗∗∗^
Road density (H6)	+	−680380.1 (339,977.2)^∗∗^	−769459 (348,453.5)^∗∗^	−195943.6 (161,600.7)	−193713 (174,378.4)
Population density (H6)	+	−< 0.001 (< 0.001)	−< 0.001 (< 0.001)	−16.489 (47.118)	−38.707 (50.25)
Income per capita (H7)	+	< 0.001 (< 0.001)	< 0.001 (< 0.001)	—	—
(Income per capita)^2^ (H7)	−	−< 0.001 (< 0.001)^∗∗∗^	−< 0.001 (< 0.001)^∗∗^	—	—
Mean household income (H7)	+	—	—	< 0.001 (< 0.001)^∗∗∗^	< 0.001 (< 0.001)^∗∗^
(Mean household income)^2^ (H7)	—	—	—	−< 0.001 (< 0.001)^∗∗^	−< 0.001 (< 0.001)
Asia population percentage	None	−0.071 (0.021)^∗∗∗^	−0.067 (0.021)^∗∗∗^	−0.024 (0.013)^∗^	−0.026 (0.014)^∗^
Hispanic population percentage	None	0.007 (0.01)	0.009 (0.011)	—	—
White people percentage	None	−0.027 (0.008)^∗∗∗^	−0.03 (0.008)^∗∗∗^	−0.007 (0.005)	−0.008 (0.005)
Regional dummy: West (controlling variable)	None	0.076 (0.369)	0.009 (0.386)	1.094 (0.312)^∗∗∗^	1.002 (0.329)^∗∗∗^
Regional dummy: South (controlling variable)	None	−0.197 (0.329)	−0.244 (0.346)	0.505 (0.306)^∗^	0.35 (0.323)
Regional dummy: Northeast (controlling variable)	None	−0.059 (0.337)	0.048 (0.36)	0.695 (0.268)^∗∗^	0.768 (0.296)^∗∗^
Total population	None	< 0.001 (< 0.001)^∗∗∗^	< 0.001 (< 0.001)^∗∗∗^	< 0.001 (< 0.001)^∗∗∗^	< 0.001 (< 0.001)^∗∗∗^
Zero-inflated part					
Intercept	None	1.871 (0.572)^∗∗∗^	1.999 (0.601)^∗∗∗^	2.749 (0.216)^∗∗∗^	3.121 (0.237)^∗∗∗^
Total population	None	−< 0.001 (< 0.001)^∗∗^	−< 0.001 (< 0.001)^∗∗^	−< 0.001 (< 0.001)^∗∗∗^	−< 0.001 (< 0.001)^∗∗∗^
Goodness of fit					
VIF		4.28	4.28	3.27	3.27
Log-likelihood		−540.3996	−489.0263	−1130.379	−992.9781
Vuong test		2.77^∗∗∗^	2.84^∗∗∗^	4.92^∗∗∗^	5.09^∗∗∗^
LR chi-square		412.5^∗∗∗^	371.24^∗∗∗^	550.12^∗∗∗^	464.77^∗∗∗^
Number of observation		3122	3122	34,413	34,413

*Note:* If the variables at the spatial unit level are not available, higher level variables were used instead. For example, there are no data on social network variables at the county–subdivision level, and the corresponding county-level data were used.

^∗^ *p* < 0.1.

^∗∗^ *p* < 0.05.

^∗∗∗^ *p* < 0.01.

**Table 14:** Diversity of the village members (total 2020 participating Villages: 88).

Member types	Mean (%)	Standard deviation (%)
Ages 64 and younger	10.94	12.24
Ages 65–74	35.00	20.58
Ages 75–84	38.20	17.92
Ages 85 and older	18.21	15.52
Impoverished (likely eligible for Medicaid or food stamps)	6.83	11.76
Economically vulnerable (not eligible for Medicaid, but struggling financially/near poor)	9.90	11.25
Have an illness, functional impairment, or disability that requires regular assistance with personal care (bathing, dressing, getting around inside the home)	10.03	14.78
Non-White (racial or ethnic minorities)	6.96	14.37
Racial or ethnic minorities: Black	5.48	14.30
Racial or ethnic minorities: Hispanic	0.97	1.67
Racial or ethnic minorities: Asian	3.28	12.06
Racial or ethnic minorities: Others	0.36	1.14

**Table 15:** Interactions between Villages and the local government.

Level of interaction (low to high: 0–5)	Content and examples of the interactions	
0	Governmental officials are aware/unaware of the presence of Villages in the jurisdiction, and little interaction exists between the local government and Villages	

1	The value of Villages is recognized by the government. The government supports the promotion of Villages and provides Villages with relevant information	Villages have publicities in the government-run media.
		The Village model and examples are mentioned in the governmental document or state act, such as the State Plan on Aging
		The government provides Villages with free vendors in certain events so they can promote themselves
		Elected officials speak highly of Villages and introduce the Village services in public settings
		Government share information with Villages

2	The government and Villages have longer and more regular interactions regarding updating information mutually and giving policy suggestions	The government actively asks for input from Villages on certain policy issues
		Governmental agencies and Villages participating in the same older adult service provider coalitions
		Some Village board members are also the volunteer members of the AAAs

3	The government provides more substantiate resources, including funds and physical spaces, to Villages to improve the well-being of older adults in the jurisdiction	Villages have the opportunity to compete and receive grants
		Villages and the government cooperate on certain projects
		The government provides Villages with physical space, such as offices or meeting rooms

4	The local government has an official whose duty is to help Villages and promote the Village concept within the jurisdiction	

5	Villages are run by a government-run organization, such as AAAs or senior centers, with the government funding a majority or the Villages' operations	

## Data Availability

The data that support the findings of this study are openly available in ResearchGate at the following website: https://www.researchgate.net/publication/379924016_Village_Survey_raw_data.

## Endnotes

^1^Village-to-Village network: https://www.vtvnetwork.org; Washington Area Villages Exchange (WAVE): https://www.wavevillages.org/

^2^Since all the coding performed by this research was carried out by one researcher, the liability adjustment is not needed. The adjustment would be required if more than one people carry out the coding.

^3^See Figure 3 for the starting years of Villages.

^4^Village point data means the Village locations.

^5^Due to this matching method, the Villages located on the border could be contained by two geographic units, thus counted twice. In fact, at the county–subdivision level, two Villages are counted twice.

^6^At the end of each section title or in the regression results table, the specific research questions or hypotheses addressed by the findings in that section are explicitly noted.

^7^The membership due information is obtained from the coalitions' website in Jan. 2022.

^8^The experience of the first Village is from *A Founder's Manual: How to Make Your Neighborhood or Town into A Village* ([43], p. 5).

^9^This fact is according to the field data obtained in a Village gathering.

^10^For regression analysis on higher incomes and very high incomes, see Section 3.2.2.3.

^11^According to the 2020 United States' Census, more than 40% of the United States' population are ethnic minorities.

^12^The website of the CCACC: https://www.ccacc-dc.org/Default_en.aspx.

^13^See Section 3.2.2.10 for the regression result.

^14^Social orientation is an attitude including postmaterialist values and altruism [50].

^15^According to a short public discussion between a speaker and several audiences at the 2002 On Aging Conference hosted by the nonprofit “Aging in America,” the people participating in the conversation thought that Villages charge high membership fees, about more than $700.

^16^In the United States, a single-house community in a higher income suburban area, such as Fairfax, Virginia, is usually considered a higher income community. This fact is also confirmed by the observation made by this study on the landscape of the community where that member lives.

^17^The benefits mentioned in this paragraph are based on the Village case data.

^18^STATA was used to conduct the regression analysis.

^19^See Section 3.1.2 for relevant case study findings.

^20^From the field observations, many people working in older adult service also thought most Villages exist in the White community; the survey result also shows that the percentage of non-White Village members is much lower than the United States' ethnic minority population, see Table 14.

^21^Many socioeconomic figures have changed during the 2002–2020 research period. Therefore, when getting independent variables, this research chose the data year closest to the year 2011 to get the average socioeconomic features of the research period. Thus, the shapefiles of the corresponding years were chosen.

^22^TIGER/Line Shapefile, 2017 [58]

^23^County subdivisions [59]

^24^General Social Survey was considered to create the social network and volunteerism variables. However, it was found that the data are not applicable, because it only has the responses from about two-hundred United States' counties. American Time Use Survey is also not applicable because the location information of the data has not been disclosed.

^25^The given definition is “professional, scientific, and management; administrative; and waste management services.”

^26^This index takes a number of factors into calculation, such as the number of various types of membership groups, turnout rate, and census response rate.

^27^Thanks to the author for providing raw data for this study.

^28^At the county level, the adjacent file for the spatial units is from [60]. At the county–subdivision level, the adjacent file is created with GeoDa and transformed with OpenRefine.

^29^Since the raw data are the shapefile of all United States' roads, this study used ArcGIS to divide the data into county and county–subdivision levels.

^30^The distribution of regional culture is based on Woodard [24].

## References

[1] Greenfield E. A., Scharlach A. E., Lehning A. J., Davitt J. K., Graham C. L. (2013). A Tale of Two Community Initiatives for Promoting Aging in Place: Similarities and Differences in the National Implementation of NORC Programs and Villages. *The Gerontologist*.

[2] Scharlach A., Graham C., Lehning A., Barrett L. L. (2012). The “Village” Model: A Consumer-Driven Approach for Aging in Place. *The Gerontologist*.

[3] Graham C. L., Scharlach A. E., Nicholson R. (2017). 2016 National Survey of US Villages. *The Center for the Advanced Study of Aging Services*.

[4] Lehning A. J., Scharlach A. E., Davitt J. K. (2017). Variations on the Village Model an Emerging Typology of a Consumer-Driven Community-Based Initiative for Older Adults. *Journal of Applied Gerontology*.

[5] Village to Village Network (2024). Village to Village Network. https://vtvnetwork.clubexpress.com/content.aspx?page_id=22%26club_id=691012%26module_id=248579.

[6] Graham C., Scharlach A. E., Kurtovich E. (2016). Do Villages Promote Aging in Place? Results of a Longitudinal Study. *Journal of Applied Gerontology*.

[7] Graham C. L., Scharlach A. E., Stark B. (2017). Impact of the Village Model: Results of a National Survey. *Journal of Gerontological Social Work*.

[8] Mair J., Martí I. (2006). Social Entrepreneurship Research: A Source of Explanation, Prediction, and Delight. *Journal of World Business*.

[9] Putnam R. D. (2001). *Bowling Alone: The Collapse and Revival of American Community*.

[10] Walker B. H., Salt D. (2006). *Resilience Thinking: Sustaining Ecosystems and People in a Changing World*.

[11] Powell W. W., Biggart N. W. (2002). Learning from Collaboration: Knowledge and Networks in the Biotechnology and Pharmaceutical Industries. *Readings in Economic Sociology*.

[12] Ganor M., Ben-Lavy Y. (2003). Community Resilience: Lessons Derived from Gilo under Fire. *Journal of Jewish Communal Service*.

[13] Administration for Community Living (2017). *A Profile of Older Americans: 2016*.

[14] William H. F. (2007). *Mapping the Growth of Older America: Seniors and Boomers in the Early 21st Century*.

[15] Laila Kearney (2018). *U.S. State Reforms Not Enough to Solve Pension Problem-Fitch*.

[16] Osborn R., Doty M. M., Moulds D., Sarnak D. O., Shah A. (2017). Older Americans Were Sicker and Faced More Financial Barriers to Health Care Than Counterparts in Other Countries. *Health Affairs*.

[17] U.S. Department of Housing and Urban Development (2017). Housing for Seniors: Challenges and Solutions. https://www.huduser.gov/portal/periodicals/em/summer17/highlight1.html.

[18] Norris F. H., Stevens S. P., Pfefferbaum B., Wyche K. F., Pfefferbaum R. L. (2008). Community Resilience as a Metaphor, Theory, Set of Capacities, and Strategy for Disaster Readiness. *American Journal of Community Psychology*.

[19] McCarthy J. D., Zald M. N. (1977). Resource Mobilization and Social Movements: A Partial Theory. *American Journal of Sociology*.

[20] Dees J. G. (1994). *Social Enterprise: Private Initiatives for Common Good*.

[21] Koe Hwee Nga J., Shamuganathan G. (2010). The Influence of Personality Traits and Demographic Factors on Social Entrepreneurship Start up Intentions. *Journal of Business Ethics*.

[22] Newman L., Dale A. (2005). Network Structure, Diversity, and Proactive Resilience Building: A Response to Tompkins and Adger. *Ecology and Society*.

[23] Stephan U., Uhlaner L. M., Stride C. (2015). Institutions and Social Entrepreneurship: The Role of Institutional Voids, Institutional Support, and Institutional Configurations. *Journal of International Business Studies*.

[24] Woodard C. (2012). *American Nations: A History of the Eleven Rival Regional Cultures of North America*.

[25] Nahemow L., Lawton M. P. (1975). Similarity and Propinquity in Friendship Formation. *Journal of Personality and Social Psychology*.

[26] Stewart J. Q. (1941). An Inverse Distance Variation for Certain Social Influences. *Science*.

[27] Coleman J. S. (1988). Social Capital in the Creation of Human Capital. *American Journal of Sociology*.

[28] Stolle D. (2007). *Social Capital*.

[29] Bigonnesse C. (2017). The Role of the Socio-Physical Environment on Aging in Place for Older Adults in Cohousing and Naturally Occurring Retirement Communities. http://summit.sfu.ca/item/17669.

[30] De Donder L., De Witte N., Buffel T., Dury S., Verté D. (2012). Social Capital and Feelings of Unsafety in Later Life: A Study on the Influence of Social Networks, Place Attachment, and Civic Participation on Perceived Safety in Belgium. *Research on Aging*.

[31] Kochera A., Straight A., Guterbock T., Krause N., Houser A. (2005). *Beyond 50.05: A Report to the Nation on Livable Communities Creating Environments for Successful Aging*.

[32] Johnson R. B., Onwuegbuzie A. J., Turner L. A. (2007). Toward a Definition of Mixed Methods Research. *Journal of Mixed Methods Research*.

[33] Maxwell J. A. (2013). *Qualitative Research Design: An Interactive Approach*.

[34] Greene J. C., Caracelli V. J., Graham W. F. (1989). Toward a Conceptual Framework for Mixed-Method Evaluation Designs. *Educational Evaluation and Policy Analysis*.

[35] Blatter P. J., Haverland D. M. (2012). *Designing Case Studies: Explanatory Approaches in Small-N Research*.

[36] Gerring J. (2009). *Case Study Research: Principles and Pratices (3rd Repr)*.

[37] Miles M. B., Huberman A. M., Saldaña J. (2014). *Qualitative Data Analysis: A Methods Sourcebook*.

[38] Saldaña J. (2016). *The Coding Manual for Qualitative Researchers*.

[39] Rupasingha A., Goetz S. J., Freshwater D. (2006). The Production of Social Capital in US Counties. *The Journal of Socio-Economics*.

[40] Positive Aging Community Director (2021). National Virtual Village Gathering 2021—Day 3 [Video Recording]. https://www.youtube.com/watch?v=4Js5uodM_eg.

[41] Tompkins E., Adger W. N. (2004). Does Adaptive Management of Natural Resources Enhance Resilience to Climate Change?. *Ecology and Society*.

[42] Positive Aging Community Director (2020). National Virtual Village Gathering 2020—Village to Village Network—Tuesday, October 6 [Video Recording]. https://www.youtube.com/watch?v=DG9kaI4lqVI.

[43] Beacon Hill Village (2006). A Founder’s Manual: How to Make Your Neighborhood or Town into A Village. https://www.beaconhillvillage.org/content.aspx?page_id=22%26club_id=332658%26module_id=346280.

[44] Wilson L. (2006). *Civic Engagement and the Baby Boomer Generation: Research, Policy, and Practice Perspectives*.

[45] Jones J. M. (2021). U.S. Church Membership Falls Below Majority for First Time. Gallup.Com. https://news.gallup.com/poll/341963/church-membership-falls-below-majority-first-time.aspx.

[46] Beacon Hill Village We Are Beacon Hill Village. https://www.beaconhillvillage.org/content.aspx?page_id=22%26club_id=332658%26module_id=344865.

[47] Foggy Bottom West End Village FBWEV Fact Sheet.

[48] Tocqueville A. de (1835). *Democracy in America*.

[49] Beacon Hill Village Meet a Member: Susan McWhinney Morse. https://www.beaconhillvillage.org/content.aspx?page_id=22%26club_id=332658%26module_id=352647.

[50] Urbano D., Ferri E., Peris-Ortiz M., Aparicio S., Peris-Ortiz M., Teulon F., Bonet-Fernandez D. (2017). Social Entrepreneurship and Institutional Factors: A Literature Review. *Social Entrepreneurship in Non-profit and Profit Sectors*.

[51] Villages NW. https://villagesnw.org/.

[52] Local Villages|Villages NW. https://villagesnw.org/nw-villages/local-2/.

[53] Villages N. W. (2015). Villages NW-Metro Board of Directors Handbook.

[54] Mcleod S. (2022). Maslow’s Hierarchy of Needs. https://www.simplypsychology.org/maslow.html.

[55] Schneider C. L. (1995). *Shantytown Protest in Pinochet’s Chile*.

[56] Swarts H. J. (2008). *Organizing Urban America: Secular and Faith-Based Progressive Movements*.

[57] Wood R. L., Fulton B. R. (2015). *A Shared Future: Faith-based Organizing for Racial Equity and Ethical Democracy*.

[58] U.S. Census Bureau (2021). TIGER/Line Shapefile, 2017. https://catalog.data.gov/dataset/tiger-line-shapefile-2017-nation-u-s-current-state-and-equivalent-national.

[59] County Subdivisions (2021). https://fedmaps.maps.arcgis.com/home/item.html?id=8e640fe423a941328039d57634c27aaa.

[60] U.S. Census Bureau (2010). County Adjacency File. https://www.census.gov/geographies/reference-files/2010/geo/county-adjacency.html#:%7E:text=The%20county%20adjacency%20file%20lists,and%20the%20U.S.%20Virgin%20Islands.

